# Differential recognition of *Haemophilus influenzae* whole bacterial cells and isolated lipooligosaccharides by galactose-specific lectins

**DOI:** 10.1038/s41598-018-34383-x

**Published:** 2018-11-02

**Authors:** Ioanna Kalograiaki, Begoña Euba, María del Carmen Fernández-Alonso, Davide Proverbio, Joseph W. St. Geme, Teodor Aastrup, Junkal Garmendia, F. Javier Cañada, Dolores Solís

**Affiliations:** 1 Centro de Investigaciones Biológicas, CSIC, Ramiro de Maeztu 9, 28040 Madrid, Spain; 2 CIBER de Enfermedades Respiratorias (CIBERES), Avda Monforte de Lemos 3-5, 28029 Madrid, Spain; 3 Instituto de Agrobiotecnología, CSIC-UPNa-Gobierno Navarra, Avda Pamplona 123, 31192 Mutilva, Spain; 4 Attana, Björnnäsvägen 21, 11419 Stockholm, Sweden; 5 Children’s Hospital of Philadelphia, Perelman School of Medicine, University of Pennsylvania, 3401 Civic Center Blvd, Philadelphia, PA 19104 United States of America; 6 Instituto de Química Física Rocasolano, CSIC, Serrano 119, 28006 Madrid, Spain

## Abstract

Bacterial surfaces are decorated with carbohydrate structures that may serve as ligands for host receptors. Based on their ability to recognize specific sugar epitopes, plant lectins are extensively used for bacteria typing. We previously observed that the galactose-specific agglutinins from *Ricinus communis* (RCA) and *Viscum album* (VAA) exhibited differential binding to nontypeable *Haemophilus influenzae* (NTHi) clinical isolates, their binding being distinctly affected by truncation of the lipooligosaccharide (LOS). Here, we examined their binding to the structurally similar LOS molecules isolated from strains NTHi375 and RdKW20, using microarray binding assays, saturation transfer difference NMR, and molecular dynamics simulations. RCA bound the LOS_RdKW20_ glycoform displaying terminal Galβ(1,4)Glcβ, whereas VAA recognized the Galα(1,4)Galβ(1,4)Glcβ epitope in LOS_NTHi375_ but not in LOS_RdKW20_, unveiling a different presentation. Binding assays to whole bacterial cells were consistent with LOS_NTHi375_ serving as ligand for VAA, and also suggested recognition of the glycoprotein HMW1. Regarding RCA, comparable binding to NTHi375 and RdKW20 cells was observed. Interestingly, an increase in LOS_NTHi375_ abundance or expression of HMW1 in RdKW20 impaired RCA binding. Overall, the results revealed that, besides the LOS, other carbohydrate structures on the bacterial surface serve as lectin ligands, and highlighted the impact of the specific display of cell surface components on lectin binding.

## Introduction

Ubiquitous in nature, carbohydrates mediate a myriad of recognition events, both in health and disease. The surface of eukaryotic cells displays a complex network of glycan structures that serve as signals in cell communication. Similarly, bacterial surfaces are profusely coated with carbohydrates, the most prominent in Gram-negative bacteria being capsular polysaccharides and lipopolysaccharides. Recognition of these structures by host receptors, including lectins of the innate immune system, can trigger immune signalling and activation, or may be exploited by the pathogen for attachment, cell entry, or host immunity subversion^1–3^. Some Gram-negative bacteria express short-chain lipopolysaccharides, referred to as lipooligosaccharides (LOSs), that mimic the carbohydrate moieties of host cells to camouflage the bacteria from the host^4^. A relevant example is *Haemophilus influenzae*, whose LOS may display the Galα(1,4)Galβ epitope characteristic of P1PK blood group antigens^5^. Expression of this disaccharide also serves to shield LOS inner core structures from recognition by naturally acquired antibodies and prevent complement- and neutrophil-mediated killing^6,7^. Of note, a hallmark of *H. influenzae* LOS is its inter- and intra-strain heterogeneity, which primarily arises from differences in the presence and phase-variable expression of biosynthetic genes and leads to variable outcomes with the host, including colonization, persistence, or acute infection^8^. Glycoproteins also decorate the surface of many bacteria, including *H. influenzae* and other important human pathogens, and different roles for the protein-linked glycans in bacterial motility, adhesion, and immune evasion or modulation have been proposed^9–12^.

**Figure 1:**
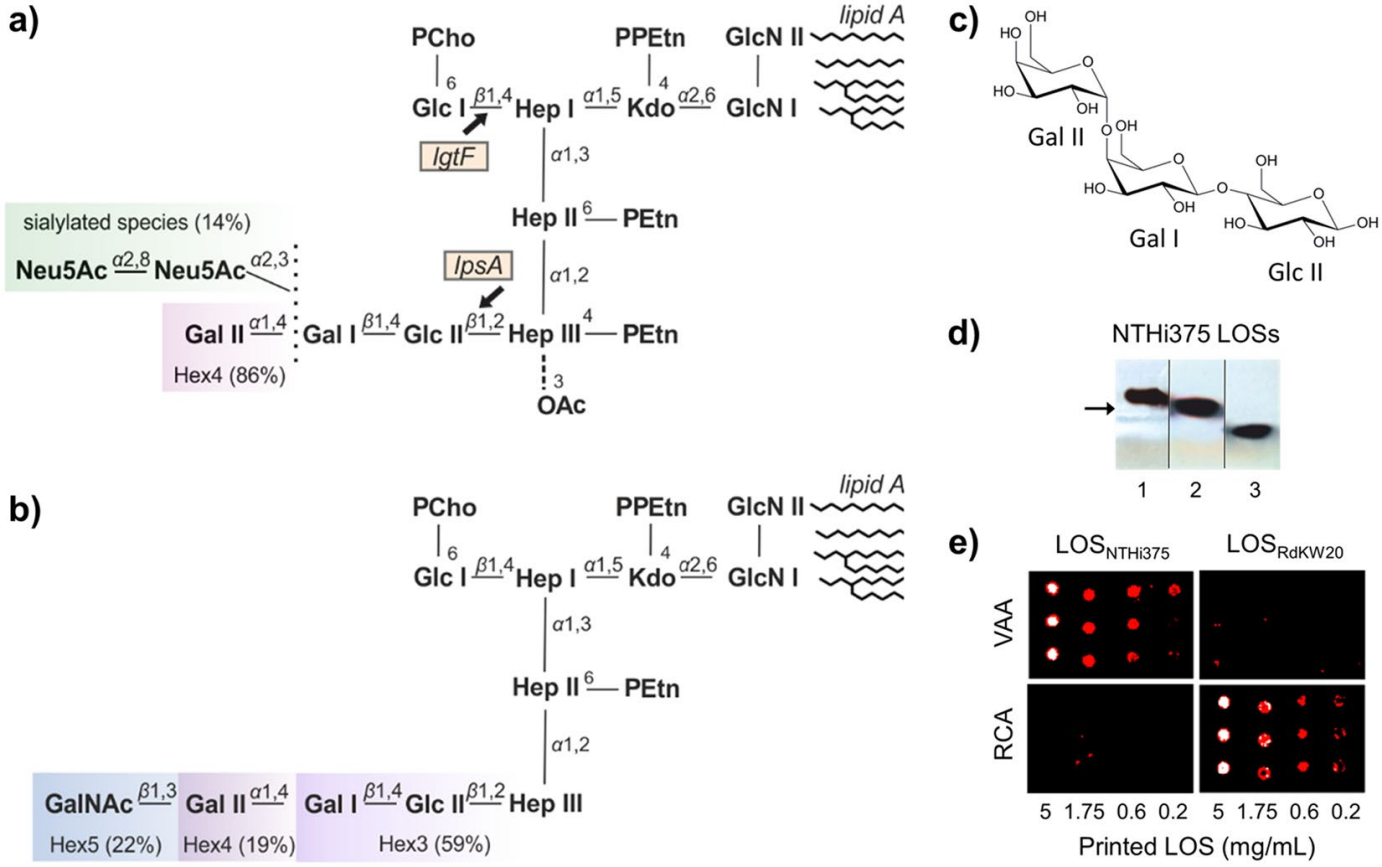
NTHi375 and RdKW20 LOS. Carbohydrate sequence and relative proportion^28,57^ of LOS glycoforms in NTHi375 (**a**) and RdKW20 strains (**b**). In panel (a), the point of action of enzymes coded for by the *lgtF* and *lpsA* genes is indicated. PEtn, phosphoethanolamine; PCho, phosphorylcholine; OAc, O-acetyl. (**c**) Structural formula of the globotriose extension at the distal mannoheptose (Hep III) of Hex4 glycoforms. (**d**) Electrophoretic mobility of LOSs isolated from NTHi375Δ*ompP5* (1), Δ*lgtF* (2), and Δ*lpsA* (3) strains (lanes cropped from the gel shown in Supplementary Fig. S2a). The arrow indicates the mobility of the Rough b-form of *Salmonella minnesota* lipopolysaccharide, used as reference (Supplementary Fig. S2a). (**e**) Binding of biotin-labelled VAA and RCA to LOS_NTHi375_ and LOS_RdKW20_ printed onto nitrocellulose-coated microarray slides as triplicates at four different LOS concentrations. Binding was detected with AF647-labelled streptavidin, as described in the Methods section.

Since the pioneer observation of Sumner and Howell that the lectin from *Canavalia ensiformis* concanavalin A (ConA) agglutinates certain bacteria^13^, agglutination by specific plant lectins has been extensively used for bacteria identification and differentiation among strains^14–18^. The underlying principle is the ability of lectins to selectively recognize particular carbohydrate structures. More recently, different lectin microarray and biosensor approaches for bacteria typing and strain discrimination have been reported^19–22^. Following the inverse strategy, we developed novel bacteria-based microarrays and quartz crystal microbalance (QCM) chips for the screening of bacterial glycosignatures, by testing the binding of a panel of lectins with diverse carbohydrate-binding specificities, and quantitative analysis of lectin−bacteria interactions^23–25^. Using this combined approach, different lectin-binding fingerprints were observed for six clinical isolates of nontypeable (non-capsulated) *H. influenzae* (NTHi), consistent with the above mentioned inter-strain heterogeneity of the bacterium^24^. An interesting finding of this study was that the galactose-specific agglutinins from *Ricinus communis* (RCA) and *Viscum album* (VAA), which show high structural homology (Supplementary Introduction and Supplementary Fig. S1), exhibited a different binding behaviour. Thus, RCA gave strong binding signals for NTHi isolates from patients with chronic obstructive pulmonary disease and from paediatric healthy carriers, whereas binding of VAA to these strains was considerably less, suggesting that the two lectins recognize different ligands on the NTHi surface. Moreover, although both RCA and VAA exhibited noticeable binding to the otitis media isolate NTHi375, indicating the availability of galactose-containing structures on the surface of this NTHi strain, LOS truncation had disparate consequences on the binding of the two lectins^24^. In particular, the LOS of this NTHi strain (Fig. 1a) is known to contain the Galα(1,4)Galβ epitope in the chain extension linked to the distal manno-heptose (Hep III) of the Hep trisaccharide inner core (Gal II-Gal I in Fig. 1a,b,c)^26,27^. The absence of this epitope in the NTHi375Δ*lic2A* mutant, lacking the glycosyltransferase that adds β-galactose (Gal I) to the glucose residue linked to Hep III (Glc II), resulted in decreased binding of VAA compared to the wild type (WT) strain, indicating that the LOS may serve as docking point for this lectin. However, no significant effect on the binding of RCA was observed, suggesting that RCA might not bind this LOS^24^.

Prompted by these findings, in this work we comparatively examined the binding of VAA and RCA to the LOS from NTHi375 and from the capsule-deficient *H. influenzae* laboratory strain RdKW20 (Fig. 1b) whose major glycoform presents terminal Galβ(1,4)Glc^28^ (Gal I-Glc II in Fig. 1a,b,c) that could potentially be recognized by the two lectins^29–31^. Microarray binding assays and saturation transfer difference (STD) NMR analysis^32^, supported by molecular dynamics (MD) simulations^33^, revealed clear-cut lectin- and LOS-specific differences in recognition. In parallel, lectin binding to NTHi375 and RdKW20 whole cells was compared using bacteria-based microarray and QCM analyses. Altogether, the results evidenced that, besides the LOS molecule, other carbohydrate structures on the bacterial surface are recognized by these two lectins, highlighting the complexity of lectin–bacteria interplays.

## Results and Discussion

### Microarray analysis of the binding of VAA and RCA to the LOS molecule from strains NTHi375 and RdKW20

Based on previous data suggesting a differential recognition by RCA and VAA of the NTHi375 LOS molecule on the bacterial surface^24^, we first examined the binding of the two lectins to the purified LOS molecule using the microarray technology. To this aim, the LOS was extracted and quantified using a combination of the Purpald assay and densitometry of LOS bands upon DOC-PAGE and silver staining (Fig. 1d, Supplementary Methods). The amount of LOS extracted from an NTHi375 mutant lacking the major outer membrane protein P5 (NTHi375Δ*ompP5*) was always significantly higher than that extracted from WT NTHi375 (Supplementary Results and Discussion and Supplementary Figure S2). Since NMR analyses proved that the structure of this LOS was identical to that reported for the WT strain^27^ (detailed in Supplementary Results and Discussion, Supplementary Figures S5–S8, and Supplementary Tables S2 and S3), for practical reasons the NTHi375Δ*ompP5*-derived LOS (hereafter referred to as LOS_NTHi375_) was used for the binding studies.

As illustrated in Fig. 1e and summarized in Table 1, strong binding signals for VAA to microarray-printed LOS_NTHi375_ were observed. Importantly, the binding was carbohydrate-mediated, as it was inhibited in the presence of lactose (above 90% of inhibition). Considering the ability of VAA to bind both α- and β-galactosides^34,35^, recognition of the major α-Gal-terminated glycoform (Hex4 according to the terminology used in Fig. 1a) could account for the binding to LOS_NTHi375_. This notion was supported by the binding behaviour of VAA towards the LOS molecules isolated from the NTHi375 mutant strains Δ*lpsA* and Δ*lgtF* (Fig. 1d and Table 1). Thus, compared to LOS_NTHi375_, VAA binding to LOS_NTHi375Δ*lpsA*_, which lacks the extension at the distal Hep (Hep III, Fig. 1a), was drastically reduced (Table 1), while binding to LOS_NTHi375Δ*lgtF*_ (lacking the extension at the proximal Hep I) was increased, hinting at a higher accessibility of the recognized epitope in the absence of the Hep I branch. Importantly, the binding of anti-lipid A antibody to the three LOSs was comparable (Table 1), indicating that the observed divergences in VAA binding were not due to a different amount of printed LOS. In striking contrast, only marginal binding of RCA to LOS_NTHi375_ was detected (Fig. 1e and Table 1). Although both VAA and RCA are galactose-specific lectins, they show differences in their fine ligand-binding specificity. In particular, RCA exhibits a clear preference for Galβ(1,4/3)GlcNAc/Glc sequences^36^. The β-Gal moiety in LOS_NTHi375_ (Gal I according to the terminology used in Fig. 1a), however, is substituted either at position 3 by sialic acid or at position 4 by α-Gal, predictably blocking RCA binding to the Galβ(1,4)Glc epitope, as these two positions are key for recognition^29,30^. Thus, only the terminal α-Gal (Gal II in Fig. 1a) could potentially be recognized by RCA. Therefore, the low affinity of this lectin for α-galactosides would explain the absence of meaningful binding signals towards LOS_NTHi375_.

**Table 1:** Binding of RCA, VAA, and anti-lipid A antibody to microarray-printed LOSs. LOSs were printed at 1 µg/mL and lectin binding was tested at a concentration of 8 µg/mL for RCA and 74 µg/mL for VAA, which gave comparable binding signals to control glycoproteins. Fluorescence intensity (relative units): +++++>40,000 >++++>20,000 >+++>10,000 >++>5,000 >+>1,000 >+/−. NT, not tested.

LOS source	VAA	RCA	Anti-lipid A antibody
NTHi375			
Δ*ompP5*	++++	+/−	+
Δ*lgtF*	+++++	NT	+
Δ*lpsA*	+	NT	+
RdKW20			
WT	+/−	+++	+

In line with this reasoning, strong binding of RCA to the LOS isolated from strain RdKW20 (LOS_RdKW20_), whose major glycoform displays terminal Galβ(1,4)Glc at the Hep III extension (Fig. 1b)^28^, was observed (Fig. 1e and Table 1). Again in striking contrast, only weak binding of VAA to LOS_RdKW20_ was detected (Fig. 1e and Table 1). This result was surprising, since ~19% of LOS_RdKW20_ bears an α-Gal-terminated Hex4 glycoform^28^ (Fig. 1b) similar to that present in LOS_NTHi375_. Therefore, an in-depth analysis of LOS_NTHi375_ and LOS_RdKW20_ epitopes recognized by VAA and RCA was clearly indicated. Accordingly, STD NMR experiments, assisted by MD simulations, were performed.

### LOS_NTHi375_ epitopes recognized by VAA

**Figure 2:**
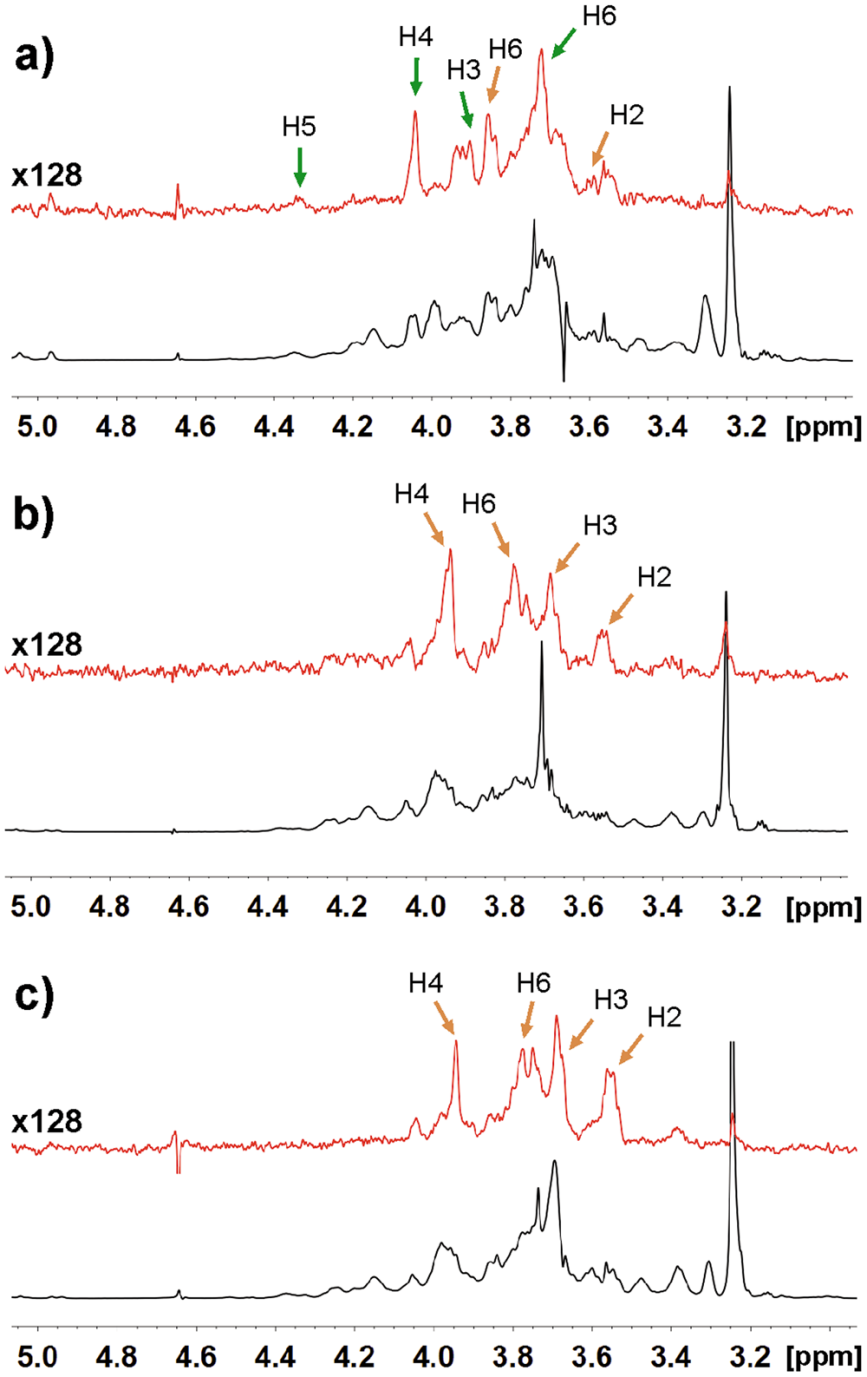
STD NMR analysis of LOS_NTHi375_ and LOS_RdKW20_ oligosaccharide epitopes recognized by VAA and RCA. STD spectra of lectin-oligosaccharide (OS) complexes (red) and reference off-resonance spectra (grey) were registered upon irradiation at 7 and 100 ppm, respectively, of 1:30 lectin:OS mixtures (8 μΜ lectin). (**a**) VAA−LOS_NTHi375_-derived OSs. (**b**) VAA−LOS_RdKW20_-derived OSs. (**c**) RCA−LOS_RdKW20_-derived OSs. Relevant STD signals are labelled. Green arrows correspond to α-Gal (Gal II) protons and orange arrows to β-Gal (Gal-I) protons.

Following assignment of the NMR resonances of the oligosaccharides (OS) released from LOS_NTHi375_ (Supplementary Information), their recognition by VAA was investigated by STD NMR (Fig. 2a). The greatest STD signals were detected for protons H3, H4, and H5 of α-Gal (Gal II), and saturation transfer to H2 and H6_A/B_ of this moiety was also observed (Table 2), indicating that this is the primary docking point for the lectin. VAA contains two different carbohydrate-binding sites, built around Trp38 and Tyr249. However, at the protein concentration used for the STD experiments VAA forms dimers in which the accessibility to the Trp-sites (located at the dimer interface) is restricted, and only the Tyr-sites are fully operative^31,37^. Focusing on this site, CH/π stacking of Gal protons H3, H4, and H5 with the phenolic ring of Tyr249 was observed in the X-ray crystal structures of VAA in complex with galactose (PDB codes 1OQL, 1PUM)^38,39^, thus fully justifying the STD signals observed.

**Table 2:** STD intensities of VAA−NTHi375Δ*ompP5* Hex4 and VAA/RCA−RdKW20 Hex3 OS complexes.

Lectin	OS glycoform	Proton	STD value	Normalized STD value^a^	STD value	Normalized STD value^a^	STD value	Normalized STD value^a^
VAA	NTHi375 Hex4		Gal II [Galα(1–4)]		Gal I [Galβ(1–4)]			
		H2	0.95%	56%	1%	56%	—	—
		H3	1.5%	83%	0.9%	50%	—	—
		H4	1.8%	100%	0.9%	50%	—	—
		H5	1.75%	95%	0.9%	50%	—	—
		H6_A-B_	1.2%	67%	1.2%	67%	—	—
VAA	RdKW20 Hex3				Gal I [Galβ(1–4)]		Glc II [Glcβ(1–2)]	
		H2	—	—	1.85%	82%	0.8%	36%
		H3	—	—	1.45%	64%	1%^c^	44%^c^
		H4	—	—	2.25%	100%		
		H5	—	—	1.2%	53%		
		H6_A-B_	—	—	1.55%^b^	69%	0.6–0.7%	27–31%
RCA	RdKW20 Hex3				Gal I [Galβ(1–4)]		Glc II [Glcβ(1–2)]	
		H2	—	—	1.95%	98%	0.55%	28%
		H3	—	—	1.7%	85%	0.8%^c^	40%^c^
		H4	—	—	2%	100%		
		H5	—	—	0.6%	30%		
		H6_A-B_	—	—	1.2%^b^	60%	0.7–0.5%	35–25%

^a^Normalized values were calculated taking the signal of Gal II H4 (NTHi375 Hex4) and Gal I H4 (RdKW20 Hex3) as 100%; ^b^Only the H6_A_ proton was assigned. ^c^Signal overlapping, the mean STD value is given. Signals of H1 protons were not quantitated as shift was close to residual HDO signal.

**Figure 3:**
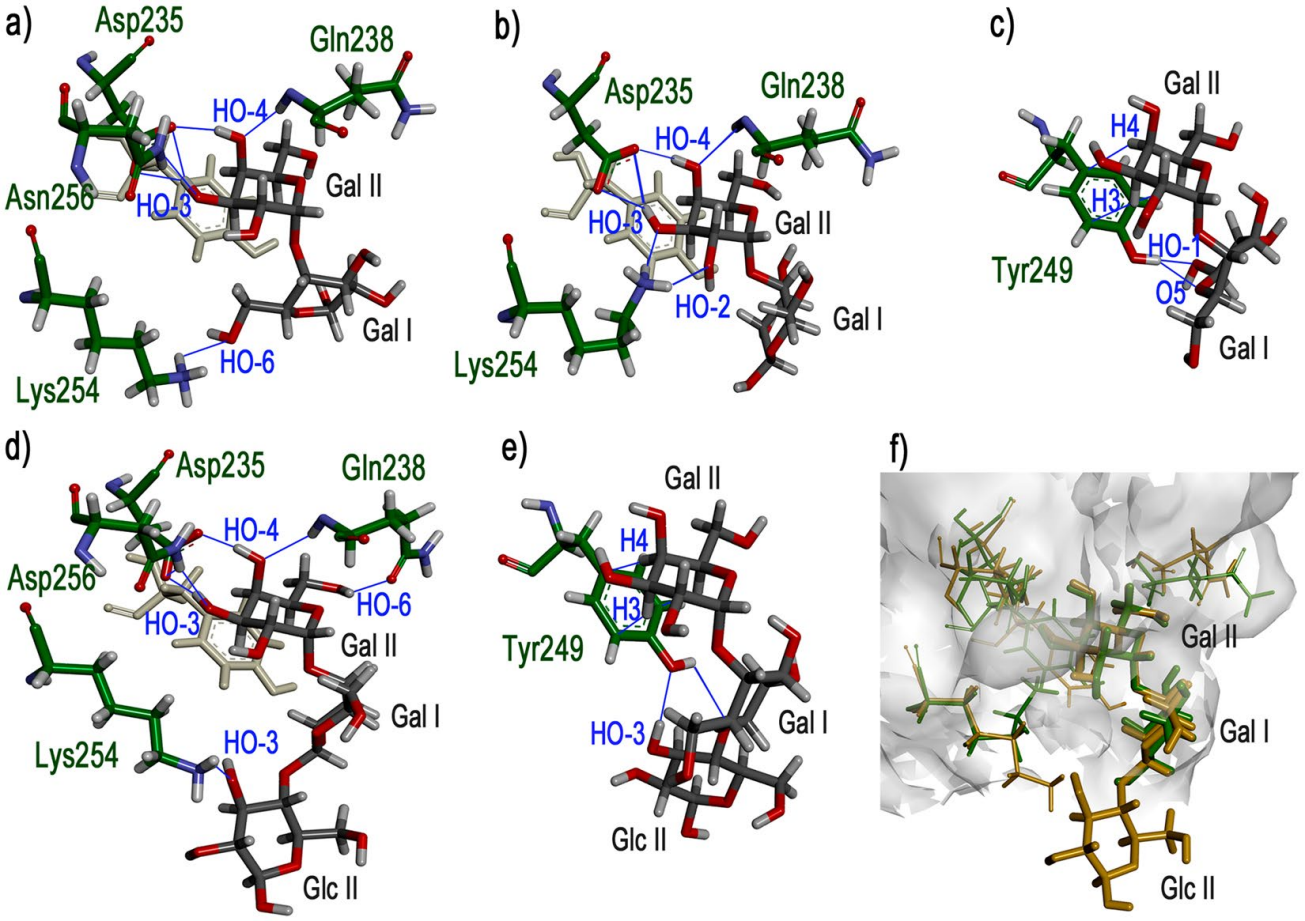
MD simulations of VAA−Galα(1,4)Galβ and VAA−Galα(1,4)Galβ(1,4)Glcβ complexes. Cluster representative structures highlighting relevant contacts detected throughout the simulations for galabiose (**a**–**c**) and globotriose (**d**,**e**) are shown. For clarity, carbon atoms of protein residues are coloured in green and those of the sugar in dark grey. Oxygen atoms are coloured in red, nitrogen in blue, and hydrogen in grey. In panels a), b), and d), Tyr249 is shown in light grey colour to serve as reference and facilitate comparison. Panel f) corresponds to a superimposition of galabiose (green) and globotriose (orange) within the binding site.

Besides Gal II, STD signals for β-Gal (Gal I) protons H2, H6_A_, and H6_B_ were detected, suggesting proximity to the protein. A previous study of the binding of the Galα(1,4)Gal disaccharide (galabiose) to VAA also detected STD signals for the two Gal residues^40^. Different binding assays with various α- and β-galactosides consistently indicated that VAA basically recognizes terminal Gal, the nature of the penultimate sugar unit and linkage configuration in general only slightly altering the binding affinity^34,35,41^. However, as a noticeable exception, a significantly higher affinity of VAA for galabiose was detected^42,43^, hinting at the establishment of additional contacts beyond the terminal α-Gal. Since the X-ray structure of VAA in complex with galabiose is not available, to clarify this issue we performed MD simulations on this lectin−sugar interaction (Fig. 3a–c).

Galα(1,4)Galβ was docked into the Tyr-site of VAA, with the α-Gal residue (equivalent to Gal-II in LOS_NTHi375_) matching the position of galactose in the crystal structure of the VAA−galactose complex. Throughout the simulations, the α-Gal residue was consistently involved in CH/π stacking with Tyr249 (Fig. 3c) and hydrogen bonding of the HO-3 and HO-4 groups with Asp235, Gln238, and Asn256 (Fig. 3a,b and Table 3), as observed in the crystal structures. In addition, transient contacts of these hydroxyl groups with other protein residues were noticed (Fig. 3b and Table 3; see Supplementary Table S4 for information on water-mediated contacts), and an interaction between HO-2 and Lys254, previously inferred from chemical mapping studies using synthetic methyl β-lactoside derivatives^35^, was also detected (Fig. 3b). More significantly, several contacts of the β-Gal residue (corresponding to Gal I in LOS_NTHi375_) were observed, the most frequent involving HO-1 and the hydroxyl group of Tyr249 (Fig. 3c and Table 3). These interactions could conceivably account for the reported higher affinity of VAA for galabiose over galactose, and were compatible with the detection of STD signals for Gal I in LOS_NTHi375_-derived OS.

**Table 3:** Direct contacts established by VAA with Galα(1,4)Galβ and Galα(1,4)Galβ(1,4)Glcβ during MD simulations.

Sugar unit	Sugar atom	Protein residue:atom	Number of clusters^a^	Mean distance (Å)	Sugar atom	Protein residue:atom	Number of clusters^a^	Mean distance (Å)
Galα(1,4)Galβ								
α-Gal	O2	Lys254:HZ1	2/26	1.86 ± 0.05				
		Lys254:HZ2	2/26	1.80 ± 0.09				
		Lys254:HZ3	4/26	1.77 ± 0.15				
	O3	Thr252:HG1	3/26	2.0 ± 0.3	H3O	Asp235:OD1	18/26	2.0 ± 0.3
		Lys254:HZ1	2/26	2.1 ± 0.4		Asp235:OD2	17/26	1.9 ± 0.2
		Lys254:HZ2	1/26	1.93				
		Lys254:HZ3	2/26	2.0 ± 0.4				
		Asn256:HD21	18/26	2.0 ± 0.3				
		Gln257:HE22	1/26	2.11				
	O4	Gln238:H	16/26	2.1 ± 0.2	H4O	Asp235:OD1	7/26	2.0 ± 0.3
		Ala239:H	2/26	1.61 ± 0.05		Asp235:OD2	24/26	1.8 ± 0.2
						Val236:O	1/26	2.42
	O6	Gln238:H	1/26	2.07	H6O	Gln238:OE1	1/26	1.92
β-Gal	O1	Tyr249:HH	7/26	1.8 ± 0.1				
	O3	Gln238:HE21	1/26	2.24				
		Gln238:HE22	1/26	1.77				
		Tyr249:HH	1/26	1.83				
	O5	Tyr249:HH	1/26	2.22				
	O6	Lys254:HZ3	1/26	2.47				
Galα(1,4)Galβ(1,4)Glcβ								
α-Gal	O2	Lys254:HZ1	2/29	2.2 ± 0.3				
		Lys254:HZ2	1/29	2.09				
		Lys254:HZ3	2/29	2.45 ± 0.05				
	O3	Lys254:HZ2	1/29	2.48	H3O	Asp235:OD1	25/29	2.1 ± 0.3
		Lys254:HZ3	1/29	2.17		Asp235:OD2	25/29	2.0 ± 0.2
		Asn256:HD21	27/29	2.1 ± 0.2				
	O4	Gln238:H	21/29	2.2 ± 0.2	H4O	Asp235:OD1	5/26	1.6 ± 0.1
		Asn256:HD21	1/29	2.38		Asp235:OD2	23/29	1.75 ± 0.1
						Val236:O	1/29	2.44
	O5	Gln238:H	1/29	2.46				
	O6	Gln238:H	1/29	2.00	H6O	Gln238:OE1	11/29	1.9 ± 0.2
		Gln238:HE21	2/29	2.47 ± 0				
β-Gal					H2O	Tyr249:OH	1/29	2.41
	O3	Gln238:HE21	3/29	2.2 ± 0.3				
β-Glc	O3	Lys254:HZ1	1/29	1.92	H3O	Tyr249:OH	2/29	2.07 ± 0.09
		Lys254:HZ2	1/29	2.20				
		Lys254:HZ3	1/29	1.97				
	O4	Tyr249:HH	1/29	2.24				

^a^Number of clusters out of 26 for Galα(1,4)Galβ and of 29 for Galα(1,4)Galβ(1,4)Glcβ in which the specified contact was detected. α-Gal H3O and H4O established at least one direct contact with ASP235:ODx in 26/26 and 29/29 clusters, respectively. Standard deviations to mean distances are given.

In the LOS molecule, Gal I is bound through a β(1,4)-linkage to Glc II (Fig. 1a). Therefore, to get further insights into the mode of binding of VAA to LOS_NTHi375_, we also performed MD simulations on the interaction of VAA with Galα(1,4)Galβ(1,4)Glcβ (globotriose, Fig. 1c). As in galabiose, the α-Gal residue was involved in CH/π interactions and hydrogen bonds of HO-3 and HO-4, together with transient contacts of HO-2 and HO-3 with Lys254 (Fig. 3d,e and Table 3). In addition, HO-6 was recurrently engaged in a strong hydrogen bond with Gln238 and several contacts of the β-Gal moiety were detected (Fig. 3d,e, Table 3, and Supplementary Table S4). Moreover, when extending from the disaccharide to the trisaccharide, different contacts of the β-Glc moiety were also detected (Fig. 3d–f, Table 3, and Supplementary Table S4). Worth mentioning, several signals in the Glc II H3/H4/H5 proton region were visible in the STD spectrum of the VAA−LOS_NTHi375_-derived OS complex, although they could not be quantitated due to extensive overlapping. Overall, the MD simulations suggested that combined interactions of VAA with the three globotriose residues appeared possible. Although the di- and tri-saccharides used for the simulations could have more conformational mobility than the intact LOS_NTHi375_ molecule, the results of the calculations were compatible with the STD experimental data and provided an explanation for the strong binding of VAA to LOS_NTHi375._

### LOS_RdKW20_ epitopes recognized by VAA and RCA

LOS_RdKW20_ bears terminal globotriose in about one fifth of its glycoform population (Fig. 1b), while its major glycoform displays terminal Galβ(1,4)Glc^28^, which could serve as ligand for both VAA and RCA. Therefore, the epitopes of LOS_RdKW20_-derived OS recognized by the two lectins were also examined by STD NMR (Fig. 2b,c).

Unexpectedly, no meaningful STD signals of α-Gal protons were detected for VAA, revealing that the galabiose epitope of the Hex4 glycoform is not recognized in this case. Interestingly, the Galα(1, 4)Gal-specific monoclonal antibody 4C4 also failed to recognize RdKW20 in colony immunoblotting and Western blot analysis^44,45^, hinting at an inappropriate presentation of this epitope for recognition. Of note, globotriose was reported to be a superior ligand for this antibody over galabiose^45^. Therefore, efficient recognition might depend on the availability of a LOS conformer that enables the establishment of 4C4 contacts with the three globotriose sugar units, and the same could apply to VAA. In this context, rotating-frame nuclear Overhauser effect (ROE) NMR studies revealed some differences between LOS_RdKW20_- and LOS_NTHi375_-derived OSs. Besides the transglycosidic ROE cross-peak for the proton pair Glc II H1/Hep III H2, common to both spectra, a ROE contact between Glc II H6 and Hep II H2 was visible for RdKW20 but not for NTHi375 OSs (Supplementary Fig. S10). Conversely, some extra ROE cross-peaks were observed for NTHi375, although they could not be identified. Thus, the conformational behaviour of the OSs seemed to differ, what could arise from the absence/presence in LOS_RdKW20_/LOS_NTHi375_, respectively, of phosphoethanolamine and/or O-acetyl substitutions at Hep III (Fig. 1a,b).

For both VAA and RCA, the strongest STD signal (Fig. 2b,c and Table 2) was observed for the H4 proton of terminal β-Gal (Gal I of the LOS_RdKW20_ Hex3 glycoform), followed by protons H2, H3, and H6_A_ of this residue. These signals were compatible with the differential contribution to the binding of the hydroxyl groups at these positions, determined by chemical mapping analyses^29,30,35^. In particular, the high contribution of the H2 proton revealed proximity to the protein, and was consistent with the proposed involvement of the HO-2 group in hydrogen bonding^35,46^. No meaningful signals for GalNAc protons were observed, in agreement with the known binding specificity of the lectins. Thus only the Galβ(1,4)Glc-terminated glycoform (Fig. 1b) of LOS_RdKW20_ was recognized by the two lectins. The low affinity of the VAA Tyr-site for lactose (*K*_a_ of 0.8–1.3 × 10^3^ M^-1^ at 25 °C)^31^ would therefore explain the weak binding of this lectin to LOS_RdKW20_. In contrast, the affinity of RCA for this disaccharide is ∼30-fold higher (*K*_a_ around 3 × 10^4^ M^-1^ at 19 °C)^47^, justifying its strong binding to this LOS. Overall, the results clearly point to a lectin- and strain-selective recognition of the *H. influenzae* LOSs, posing the question on the impact of this behaviour on the binding to the entire bacteria. Therefore, we examined the binding of VAA and RCA to the bacterial surface using our combined bacteria-based microarray and QCM approach.

### Binding of VAA and RCA to NTHi375 and RdKW20 cells

Four strains were selected for the analysis. Besides NTHi375, lectin binding to its Δ*ompP5* mutant, which displays a higher amount of LOS on its surface, was examined. Regarding RdKW20, another distinctive feature of this strain is the absence of the high molecular weight adhesin HMW1, which is expressed by NTHi375 and is glycosylated at multiple asparagine residues with N-linked Gal/Glc monosaccharides or Glc-Glc/Gal-Glc disaccharide units^48^. As these Gal/Gal-Glc moieties could potentially serve as ligands for VAA and/or RCA, we also included in the analysis a transformed RdKW20 strain expressing the HMW1 adhesin of NTHi strain 12 (designated as RdKW20*hmw1*_*strain12*_)^49,50^, which shares 93% homology with HMW1 from NTHi375.

**Figure 4:**
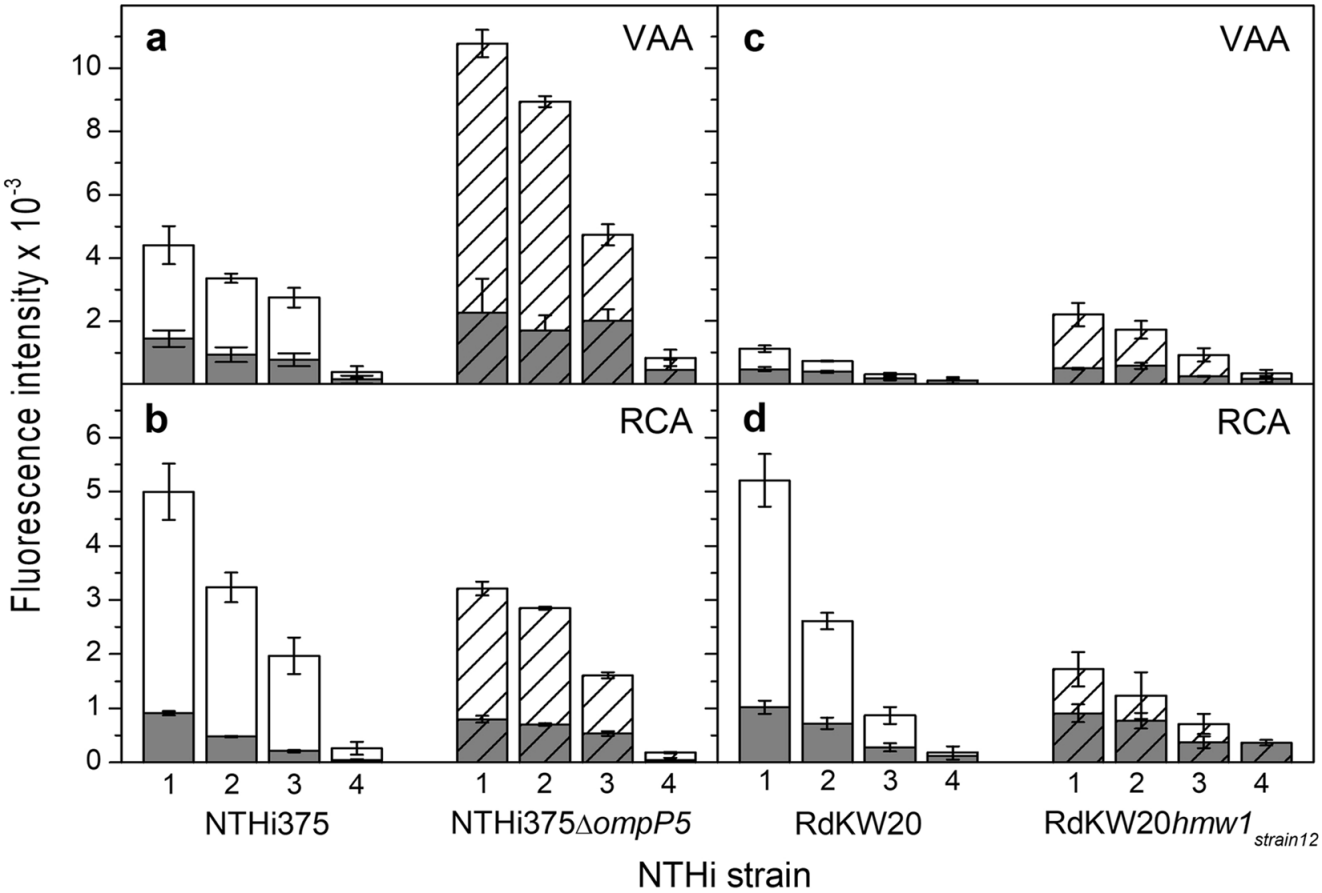
Microarray analysis of the binding of VAA and RCA to NTHi375 and RdKW20 cells. Bacteria were printed as triplicates at four different dilutions (1–4, corresponding to OD_600_ of 1, 0.6, 0.3 and 0.1, respectively) and binding of biotin-labelled VAA (**a**,**c**) and RCA (**b**,**d**) was assayed in the absence (white columns) and presence (grey columns) of 0.1 M lactose. Lectin binding was determined by incubation with AF647-streptavidin. Data shown correspond to the mean of at least two different experiments. Error bars indicate the standard deviation to the mean. (**a**,**b**) NTHi375 WT (unpatterned columns) and Δ*ompP5* mutant (dashed columns). (**c**,**d**) RdKW20 WT (unpatterned columns) and transformed *hmw1*_*strain12*_ strain (dashed columns).

Starting with the microarray analysis, significant binding of both VAA and RCA to NTHi375 was observed (Fig. 4a,b), as previously reported^24^. However, the two lectins exhibited a disparate behaviour towards the Δ*ompP5* mutant. Thus, VAA binding signals increased noticeably compared to the WT strain (Fig. 4a), indicating that LOS_NTHi375_ serves as docking point for this lectin on the bacterial surface. In contrast, binding of RCA to the Δ*ompP5* mutant was less (Fig. 4b), suggesting that the availability and/or accessibility of RCA ligand(s) is reduced upon LOS overexpression. When testing the RdKW20 strain (Fig. 4c,d), binding signals for RCA were comparable to those observed for NTHi375, whereas for VAA they were perceptibly weaker. Keeping in mind that NTHi375 and RdKW20 strains have a distinct genetic background, the weaker binding of VAA could be explained, at least in part, by the weak recognition of LOS_RdKW20_, as opposed to the strong binding to LOS_NTHi375_. The absence of HMW1 on the RdKW20 surface could also have a bearing on the observed behaviour. Indeed, VAA binding to RdKW20*hmw1*_*strain12*_ was noticeably stronger than to WT RdKW20 (Fig. 4c), suggesting that the Gal moieties decorating HMW1_*strain12*_ may serve as docking sites for this lectin. Again in striking contrast, the binding of RCA to the transformed RdKW20 strain was significantly weaker (Fig. 4d), implying that the HMW1 glycoprotein is not a ligand in this case. Moreover, expression of HMW1 at the RdKW20 surface apparently decreased the availability of ligands for RCA, as similarly observed for NTHi375Δ*ompP5*. Thus, binding of RCA to NTHi375 seems to involve recognition of sugar epitopes other than those displayed by HMW1 and the LOS. The HMW2 glycoprotein, which is highly homologous to HMW1 (71% identity, 80% similarity)^51,52^, could be a possible ligand candidate, but other alternatives, as e.g. recognition of a so far unidentified glycoprotein or of a small glycolipid cannot be excluded, warranting further study.

The kinetic parameters and affinity of lectin binding to the bacteria were next examined by QCM. Although the results of the microarray analysis pointed to the likely presence of more than one ligand on the bacterial surface, the use of 1:2 models did not significantly improve the quality of the fits to experimental data over those obtained with a 1:1 model. Therefore, the latter was used in all cases as the simplest approximation to determine the overall binding parameters (Table 4), as previously done for the binding of RCA to NTHi375^24^. The results here obtained for this bacterium−lectin pair were comparable to those reported before, particularly considering that the extremely slow dissociation rate, in the limit of detection of the technique, makes difficult a precise quantitation of the *k*_d_^24^. Similar parameters were found for the binding of RCA to NTHi375Δ*ompP5*, what could be consistent with the recognition of the same ligand(s) in the WT and mutant strains. An analogous behaviour was observed for VAA, although for this lectin dissociation was three orders of magnitude faster, resulting in a proportionally lower binding affinity towards the two strains. The analysis of RCA and VAA binding to RdKW20 and RdKW20*hmw1*_*strain12*_ yielded the same picture, with comparable parameters determined for the binding of each lectin to both strains. Thus, the absence/presence of HMW1, which according to the microarray analysis appeared to serve as ligand for VAA, did not appreciably alter the overall binding kinetics. Moreover, the presence of unidentified ligand(s) for VAA on the RdKW20 surface could be inferred, as similarly reasoned to explain the binding of RCA to NTHi375.

**Table 4:** QCM analysis of the binding of RCA and VAA to NTHi strains. Association rate (*k*_a_), dissociation rate (*k*_d_) and dissociation constants (*K*_D_) derived from the fitting of experimental data using a 1:1 binding model. Molar concentrations were calculated considering molecular masses of 120 kDa for RCA and 114 kDa for VAA. Standard deviations to mean values are given.

Lectin	Parameter	Strain			
		NTHi375	NTHi375 Δ*ompP5*	RdKW20	RdKW20 *hmw1*_*strain12*_
RCA	*k*_a_ (M^−1^ s^−1^)	2.15 ± 0.01 × 10^5^	6.49 ± 0.01 × 10^5^	6.47 ± 0.01 × 10^5^	2.70 ± 0.01 × 10^5^
	*k*_d_ (s^−1^)	1.3 ± 0.1 × 10^−7^	1.5 ± 0.1 × 10^−7^	9.55 ± 0.03 × 10^−7^	2.9 ± 0.5 × 10^−7^
	*K*_D_ (M)	5.9 ± 0.9 × 10^−13^	2.2 ± 0.3 × 10^−13^	1.48 ± 0.01 × 10^−12^	1.1 ± 0.1 × 10^−12^
VAA	*k*_a_ (M^−1^ s^−1^)	6.29 ± 0.01 × 10^5^	8.60 ± 0.01 × 10^5^	6.71 ± 0.01 × 10^5^	4.42 ± 0.01 × 10^5^
	*k*_d_ (s^−1^)	2.42 ± 0.03 × 10^−4^	5.4 ± 0.1 × 10^−4^	4.37 ± 0.02 × 10^−4^	3.39 ± 0.07 × 10^−4^
	*K*_D_ (M)	3.84 ± 0.04 × 10^−10^	6.3 ± 0.1 × 10^−10^	6.50 ± 0.03 × 10^−10^	7.7 ± 0.2 × 10^−10^

### Conclusions

A comparative analysis of the binding of RCA and VAA to NTHi375 and RdKW20 cells and to the respective isolated LOS molecules revealed that, besides the LOS, other carbohydrate structures on the bacterial surface serve as efficient ligands for these lectins. It seems reasonable to presume that this may also be the case for other lectins, including those of the innate immune system. Combined, bacterial carbohydrates build complex cell surface sceneries, which are further defined by the relative abundance, accessibility, and specific presentation of the different components. A meaningful example is the decreased binding of RCA to NTHi375Δ*ompP5* and RdKW20*hmw1*_*strain12*_ compared to the respective WT strains, apparently resulting from (over)expression of the LOS and HMW1, respectively. Altogether, the results stressed the importance of examining lectin binding to entire bacterial cells.

Nevertheless, analysis of the binding to isolated bacterial components is evidently very helpful for identification of ligand candidates and discovery of unforeseen factors affecting recognition. This is the case for the Hex4 glycoform of LOS_RdKW20_, which, contrary to expectations, was not bound by VAA possibly due to an inappropriate presentation of the globotriose epitope. This presentation is also likely responsible for the undetectable binding of the Galα(1,4)Gal-specific antibody 4C4 to this strain^44,45^. Of note, the weak binding of VAA to NTHi strains 398 and 1566 previously observed^24^ also correlated with a lack of reactivity of 4C4 towards these strains^53^. It is tempting to speculate that phosphoethanolamine and/or O-acetyl substitutions at the distal Hep III residue could have a bearing on the conformational preferences of this branch, with consequences on the recognition by galabiose/globotriose-binding proteins. These substitutions are frequently present in the LOS of NTHi and other bacterial species. A correlation between the presence of phosphoethanolamine and bacterial resistance to antimicrobial peptides or to complement-mediated killing has been established^54,55^, and, in *H. influenzae*, O-acetylation is a phase-variable trait whose expression is modulated by environmental conditions, conferring resistance to complement-mediated killing^56^. We hypothesize that these substitutions could serve as molecular switches for hiding/exposing particular carbohydrate epitopes, such as the host-like Galα(1,4)Galβ epitope characteristic of P1PK blood group antigens.

Of general significance, the results here reported for NTHi reveal that a lack of reactivity of a specific antibody or lectin towards isolated bacterial components or entire cells should not necessarily be interpreted as the absence of the recognized epitope, an observation that could be extrapolated to other bacterial strains and species. What is more, hiding/exposure of carbohydrate epitopes could be a mechanism exploited by certain bacteria to control recognition by host receptors and, thereby, modulate the outcome of the host-pathogen interplay at the infection niche.

## Methods

### Microarray binding assays

Probes, including SYTO-13-labelled bacteria suspensions (OD_600_ from 0.1 to 1) and purified LOS (0.03–1 mg/mL), were printed as triplicates in a dose-response format on 16-pad nitrocellulose-coated glass slides, using a non-contact Arrayjet Sprint Inkjet Microarrayer, as described^24^. To enable post-array monitoring of the spots, the Cy3 fluorophore (GE Healthcare) was added to the LOS solutions at 1 μg/mL final concentration^23^. The arrays were scanned for SYTO-13 and Cy3 signals, as described^23^.

To test the binding of anti-lipid A antibody to printed LOSs, slides were blocked for 1 h with 5 mM sodium phosphate buffer, pH 7.2, 0.2 M NaCl (PBS), containing 2% bovine serum albumin and 0.25% Tween 20 (both from Sigma-Aldrich), and next washed with PBS containing 0.05% Tween 20 (washing buffer). Slides were subsequently incubated for 1 h with a 1:500 dilution of goat polyclonal anti-*E. coli* O157-lipid A antibody (Abcam plc) in PBS containing 1% bovine serum albumin and 0.1% Tween 20 (overlay buffer). After three short washes with washing buffer, slides were incubated for 45 min with a 1:1,000 dilution of biotinylated rabbit anti-goat IgG (Abcam plc) in overlay buffer. Following three additional washes, slides were finally incubated for 35 min with AlexaFluor-647 (AF647)-labelled streptavidin (Invitrogen) at 1 μg/mL in overlay buffer. Finally, slides were thoroughly washed with washing buffer, PBS, and milli-Q water and dried under a nitrogen stream. All incubation steps took place in a dry, dark place, at 20 °C.

The binding of biotin-labelled lectins to bacteria and LOS microarrays was tested similarly, except that bovine serum albumin was not included in the blocking and overlay buffers. Following blocking and washing, slides were incubated for 75 min with the lectins in PBS containing 0.1% Tween 20, in the absence or presence of 0.1 M lactose. Then, the slides were washed and incubated with AF647-labelled streptavidin as described above.

### STD NMR experiments

STD experiments were performed with LOS_NTHi375_- and LOS_RdKW20_-derived soluble oligosaccharides (see Supplementary Information) using an oligosaccharide:protein molar ratio of 30:1. Selective saturation of the protein was achieved by using a train of 40 Gaussian-shaped pulses of 50-ms each, separated by a 1-ms delay (approximate saturation time of 2 s). The residual HDO signal was suppressed by hard-pulse gradient tailored excitation (WATERGATE) or gradient-based water suppression pulse (*esgp*), and background protein resonance signals were subtracted. An off-resonance frequency of δ = 100 ppm and on-resonance frequency of δ = 7 ppm were applied, targeting a spectrum region where no signals are observed for the ligand. To exclude non-specific saturation of oligosaccharide protons upon irradiation, control experiments were performed in the absence of lectin. The highest STD signal was taken as 100% and other STD values were normalized with respect to this signal.

### Molecular dynamics simulations

Galα(1,4)Galβ and Galα(1,4)Galβ(1,4)Glcβ structures were built and docked into the Tyr-site of the crystal structure of the VAA−galactose complex (PDB code 1OQL), and the respective complexes were processed to get the proper input files for MD simulations with AMBER 12, as detailed in the Supplementary Information. Frames of MD simulations were analysed for robustness and equilibrium throughout the simulations, and conformationally clustered. The most representative structure for each cluster was selected for discussion.

### QCM kinetic and affinity studies

Bacteria chips were prepared by capturing bacteria cells onto ConA-derivatized LNB (Low Non-specific Binding) surfaces (Attana AB), as described previously^24^. The binding analysis was performed by consecutive duplicate injections of increasing concentrations of RCA or VAA (1.5–5 µg/mL) for 84 s, each followed by injection of running buffer for 600 s. The surfaces were regenerated after each cycle using one 30-s pulse injection of 10 mM glycine, pH 1.2, 0.5 M NaCl, and immediately re-equilibrated with running buffer. Data were collected using Attester software (Attana AB) and analysed with Evaluation software (Attana AB) and TraceDrawer (Ridgeview). In all cases, the signal obtained from the reference chip surface was subtracted from the sensograms obtained for the ConA-bacteria surfaces.

## Electronic supplementary material

Supplementary information

## Data Availability

Data generated in this study are available from the corresponding author on request.
