# Supplementary material for: Differential recognition of Haemophilus influenzae whole bacterial cells and isolated lipooligosaccharides by galactose-specific lectins
Source: Sci Rep. 2018 Nov 2;8:16292. doi: 10.1038/s41598-018-34383-x (PMC6215012; doi:10.1038/s41598-018-34383-x)
Supplement: Supplementary file 1 — Supplementary information [file 41598_2018_34383_MOESM1_ESM.pdf]

Supplementary information

Differential recognition of *Haemophilus influenzae* whole bacterial cells and isolated lipooligosaccharides by galactose-specific lectins

Ioanna Kalograiaki[1,2], Begoña Euba[2,3], María del Carmen Fernández-Alonso[1], Davide Proverbio[4], Joseph W St Geme III[5], Teodor Aastrup[4], Junkal Garmendia[2,3], F. Javier Cañada[1] & Dolores Solís*[2,6]

[1]Centro de Investigaciones Biológicas, CSIC, Ramiro de Maeztu 9, 28040, Madrid, Spain

[2]CIBER de Enfermedades Respiratorias (CIBERES), Avda Monforte de Lemos 3-5, 28029 Madrid, Spain

[3]Instituto de Agrobiotecnología, CSIC-UPNa-Gobierno Navarra, Avda Pamplona 123, 31192 Mutilva, Spain

[4]Attana, Björnnäsvägen 21, 11419 Stockholm, Sweden

[5]Children's Hospital of Philadelphia, Perelman School of Medicine, University of Pennsylvania, 3401 Civic Center Blvd, Philadelphia, PA 19104, United States of America

[6]Instituto de Química Física Rocasolano, CSIC, Serrano 119, 28006 Madrid, Spain

*Email: d.solis@iqfr.csic.es

Supplementary Introduction

The galactose-specific agglutinins from *Ricinus communis* (RCA) and *Viscum album* (VAA) belong to the family of AB-type ribosome-inactivating proteins[1], which consist of two chains linked by a disulfide bridge: an A chain with rRNA *N*-glycosidase activity and a B chain with carbohydrate-binding activity. VAA contains two carbohydrate-binding sites per B-chain, which are characterized by the central positioning of the aromatic ring of Trp38 and Tyr249, respectively. At submicrogram-per-mL concentrations VAA forms $[AB]_2$ dimers through contacts of adjacent B chains (Fig. S1a). Dimerization spatially restricts accessibility to the Trp-sites, so that only the Tyr-sites are fully operative in the dimer[2]. On the other hand, RCA forms dimers through contacts of the A (Fig. S1b)[3]. The Trp sites are fully exposed in the RCA dimers, but the second set of carbohydrate-binding sites in the B chains is not functional due to a mutation introducing a histidine residue instead of Tyr. Thus, the ligand binding ability of the VAA and RCA dimers resides in the Tyr sites and Trp sites, respectively. As observed in the X-ray crystal structures of the agglutinin–galactose complexes, besides stacking interactions with the aromatic rings the two sets of sites share strong hydrogen bonds of the side chains of asparagine and aspartic acid residues (Asn256/Asp235 and Asn46/Asp22, respectively) with the hydroxyl groups at positions 3 and 4 of galactose (Fig. S1c,d). The RCA Trp site, however, exhibits a more complex hydrogen bonding network, including a contact of Glu26 with the hydroxyl at position 1 in β configuration. This contact is not possible in the VAA Tyr site due to the presence of an alanine residue (Ala 239) in the equivalent position.

Supplementary Results and Discussion

Isolation of $LOS_{NTHi375}$. The LOS of NTHi375 was extracted and quantified using a combination of the Purpald assay[4] and densitometry of LOS bands upon DOC-PAGE and silver staining, using an optimized protocol (see Supplementary Methods for experimental details) that enabled detection and reliable quantitation of LOS bands of (or even below) 100 ng (Fig. S2a). The yield of LOS extracted from wild type (WT) NTHi375 was insufficient to perform microarray and NMR experiments at the LOS concentrations required. Therefore, strain NTHi375Δ*ompP5*, which lacks the major outer membrane protein P5 and keeps unaltered the enzymatic machinery involved in LOS biosynthesis, was used as source for LOS isolation. This mutant rendered high signal intensity upon bacterial colony immunoblot analysis using the anti-phosphorylcholine (PCho) monoclonal antibody TEPC-15. Given that incorporation of PCho to the LOS molecule is phase variable, this observation suggests that NTHi375Δ*ompP5* is mostly PCho phase ON, although a higher LOS production in this mutant contributing to the increased TEPC-15 signal cannot be completely excluded[5,6]. Indeed, although batch-to-batch variations in the yield were observed, the amount of LOS extracted from NTHi375Δ*ompP5* was always significantly higher than that extracted from a WT NTHi375 culture of equal biomass (Fig. S2a,b), directly pointing to a higher LOS abundance in this mutant, maybe due to compensatory changes in the bacterial surface upon inactivation of the *ompP5* gene. The structure of the NTHi375Δ*ompP5*-derived LOS (hereafter referred to as $LOS_{NTHi375}$) was characterized by NMR spectroscopy and found to be identical to that reported for the WT strain, as described in the next section.

NMR structural characterization of isolated LOSs. The ^{1}H-NMR spectra of isolated LOS_{RdKW20} and $LOS_{NTHi375}$ in D_2O showed only broad undefined signals, most probably due to lipid-mediated aggregation, as previously reported for LOS_{RdKW20}[7]. To overcome this problem, the linkage between 3-deoxy-D-manno-oct-2-ulosonic acid (Kdo) and lipid A was hydrolysed under mild acid conditions, yielding insoluble lipid A and soluble oligosaccharides (OSs) derived from the various LOS glycoforms. Well defined resonances were observed for the OSs, particularly in the anomeric proton

region (4.4-5.7 ppm) of the spectrum. A collection of ^{1}H-^{13}C HSQC, TOCSY, and ROESY experiments (Supplementary Fig. S3-10) helped to the resolution of signals overlapping in the ^{1}H dimension, and enabled assignment of chemical shifts and coupling constants of the anomeric protons. The results obtained for LOS_{RdKW20}-derived OSs (Fig. S3 and S4, and Table S1) were comparable to those previously reported for this strain[7], confirming the presence of three main glycoforms bearing terminal β-Gal (Hex3), α-Gal (Hex4), or β-GalNAc (Hex5) at the Hep III branch. Regarding $LOS_{NTHi375}$-derived OSs (Fig. S5-S8, and Tables S2 and S3), anomeric resonances were observed in the ^{1}H-NMR spectrum at δ = 5.03-5.13 and 5.60-5.68 ppm, corresponding to the three Hep residues of the inner core. Their identities were confirmed by ROE cross-peaks between the respective H1-H2 intra-residue pairs (Table S3). Intense transglycosidic ROE connectivities between Hep III H1/Hep II H2 and Hep II H1/Hep I H3 (Fig. S7 and Table S3) were in agreement with the sequence of the inner-core trisaccharide being L-α-D-Hep-(1,2)-L-α-D-Hep-(1,3)-L-α-D-Hep-(1→). Acetylation at position 3 of Hep III was inferred from the low field shift of Hep III H3[8]. The anomeric signal at 4.47 ppm was assigned to the terminal β-Gal (Gal I) of a Hex3 glycoform, while the anomeric signal at 4.97 ppm, which exhibited a small $J_{1,2}$ value (3.7 Hz), was assigned to the terminal α-Gal (Gal II) of a Hex4 glycoform (Table S2). ROE cross-peaks between Gal I H1/Glc II H3/H4/H5 and Gal II H1/Gal I H4/H6 (Fig. S8) were in agreement with the presence of Galβ(1,4)Glcβ and Galα(1,4)Galβ(1,4)Glcβ extensions at Hep III of Hex3 and Hex4 glycoforms, respectively. Based on the relative ^{1}H-^{13}C HSQC intensities of the anomeric signals of terminal Gal I and Gal II in these two forms, a Hex3:Hex4 1:4 proportion was roughly estimated. Using electrospray-ionisation mass spectrometry[9], the NTHi375 LOS was previously described to be predominantly composed of Hex4 glycoforms, bearing a Galα(1,4)Galβ(1,4)Glcβ extension at Hep III, together with mono- and di-sialylated Hex3 species accounting respectively for 9% and 5% of the LOS. However, no OS-bound Neu5Ac was detected in our NMR analysis. A plausible explanation is that the conditions used for mild acid hydrolysis of the Kdo–lipid A bond could also result in cleavage of the acid-labile Neu5Ac linkages[10,11]. Therefore, it seems reasonable to presume that the Hex3 form here observed for $LOS_{NTHi375}$-derived OSs resulted from hydrolysis of sialylated species. The small difference in the estimated population of Hex3 compared to that reported for sialylated NTHi375 LOS species could be attributed to the different approaches used in each case. A close inspection of the STD spectrum of VAA in complex with $LOS_{NTHi375}$-derived OSs hinted at protons of the terminal β-Gal of the Hex3 glycoform suffering saturation transfer. However, as this glycoform is not expected to be naturally present in $LOS_{NTHi375}$, these signals were ignored. Overall, comparison of the NMR structural information obtained for the NTHi375*ΔompP5* OSs with the mass spectrometry-based structure reported for WT NTHi375 LOS[9] was consistent with the mutant and WT strains bearing identical LOSs.

Supplementary Methods

Lectins. RCA and ConA were purchased from Vector Laboratories. VAA was isolated from *Viscum album* extracts, as previously described[12]. The concentration of RCA and ConA was determined from the absorbance at 280 nm, using the extinction coefficient calculated from the amino acid sequence with the ProtParam tool, available at http://web.expasy.org/protparam. VAA concentration was determined by the Lowry assay using ConA as standard[13]. When required, RCA and VAA were biotinylated by incubation for 1 h at 20 ºC with biotinamidocaproate ester derivative (GE Healthcare Life Sciences), according to the manufacturer's recommendations. To prevent modification of residues of the carbohydrate-binding site, biotinylation was performed in the presence of 20 mM lactose.

Bacterial strains and isolation of LOS. NTHi strains used in this study included the otitis media isolate NTHi375, its isogenic mutants Δ*ompP5* (lacking the major outer membrane protein P5), Δ*lgtF*

(whose LOS lacks the extension at Hep I), and Δ*lpsA* (lacking the LOS extension at Hep III), the laboratory strain RdKW20, and RdKW20$hmw1_{strain12}$, a transformed RdKW20 strain carrying the *hmw1* operon of NTHi strain 12. Bacteria were grown on chocolate agar and brain-heart infusion medium supplemented with 10 µg/mL hemin and 10 µg/mL *β*-nicotinamide adenine dinucleotide (sBHI), fixed with 4% paraformaldehyde, and labelled with SYTO-13 as described previously[14].

For LOS isolation, NTHi strains were inoculated (3-5 colonies) in 400 mL sBHI and incubated at 37 °C and 5% CO_2 under shaking at 180 rpm. After 12 h, OD_{600} was measured and the viability tested by serial dilution and plating on sBHI agar. Bacteria pellets were collected by centrifugation at 6,000 × *g* for 15 min at 4 ºC, resuspended in water (33.33 mL water/g of dry pellet), and thoroughly mixed with an equal volume of 66 ºC-preheated equilibrated phenol solution (Sigma-Aldrich). After incubation for 15 min at 66 ºC, the phenol-bacteria suspension mixture was cooled on ice and centrifuged at 8,000 × *g* for 15 min at 4 ºC for separation of the aqueous and phenol layers. The phenol layer was discarded and the LOS was precipitated from the aqueous layer by addition of four volumes of methanol containing 1% sodium acetate-saturated methanol and incubation for at least 12 h at -20 ºC. The precipitate was recovered by centrifugation at 8,000 × g for 15 min at 4 ºC, resuspended in 5 mL water and exhaustively dialysed against distilled water. The dialysate was then centrifuged at 100,000 × g for 6 h at 4 ºC, and the resulting pellet was resuspended in 2 mL water and lyophilised. For elimination of contaminating nucleic acids, the lyophilisate was resuspended at 10 mg/mL in 100 mM Tris-HCl, pH 7.0, containing 0.05% NaN_3, and digested with 50 µg/mL DNAse II and RNAse I (Sigma-Aldrich) for 30 min at 37 ºC. Next, protein traces were digested by incubating thrice with 50 µg/mL of proteinase K (Sigma-Aldrich) for 3 h at 55 ºC. The LOS was then precipitated and recovered by centrifugation as described above, and the pellet was resuspended in 2 mL water and exhaustively dialysed against distilled water. The dialysate was centrifuged at 100,000 × g for 6 h at 4 ºC, and the resulting pellet containing purified LOS was resuspended in 200 µL of distilled water. The purity of LOS suspensions was assessed by polyacrylamide gel electrophoresis in the presence of sodium deoxycholate (DOC-PAGE) and silver staining, as described below. LOS concentration was determined colorimetrically by the Purpald assay[4,15], using Kdo (Sigma-Aldrich) and L-glycero-D-manno-heptose (Carbosynth) as standards, and by densitometric quantitation of electrophoretic LOS bands.

Sodium deoxycholate-polyacrylamide gel electrophoresis (DOC-PAGE) and silver staining. Tricine-DOC-PAGE was carried out using a discontinuous system consisting in 1) a separating gel containing 6 M urea and composed of 16.5% total acrylamide (T), with a concentration of 6% bisacrylamide (C) relative to the total concentration, 2) a spacer gel composed of 10% T and 3% C, and 3) a stacking 4% T/3% C gel, essentially as described[16], except that the gels contained 0.15% DOC instead of SDS, and the sample buffer was 0.1 M Tris-HCl, pH 6.8, containing 1% DOC (w/v), 20% glycerol, and 0.1% bromophenol blue. Samples were mixed 1:1 with sample buffer and loaded into the gels without previous boiling.

For silver staining of LOS bands, the protocol described by Schägger[17] was optimized for oligosaccharide detection. First, the gel was soaked in fixing solution composed of methanol/acetic acid/milli-Q water (40:10:50 v/v/v) for 45 min. As crucial step, the gel was next soaked for 10 min in fixing solution containing 0.7% periodic acid for LOS oxidation. After thorough washing with milli-Q water, the gel was sensitized by incubation in 0.02% sodium thiosulfate for 1 min. Following two 1-min washes with milli-Q water for removing excess ions, the gel was incubated for 45 min at 4 ºC with staining reagent containing 0.1% silver nitrate and 0.028% formaldehyde. After a brief wash with milli-Q water, developer solution consisting in 0.018% formaldehyde, 3% potassium carbonate, and 0.001% sodium thiosulfate was added. Development was stopped when considered appropriate by addition of 5% acetic acid. Gels were thoroughly washed with milli-Q water and stored in 7% acetic

acid at 4 ºC. For band quantitation, gels were scanned with an Epson Perfection 3200 Photo scanner and the images digitized using the UN-SCAN-IT-gel v6.1 software (Silk scientific). The Rb-form of the lipopolysaccharide from *Salmonella minnesota* (Enzo Life Sciences) was used as reference for comparison of band mobilities.

NMR experiments. Two mg of NTHi375Δ*ompP5*- or RdKW20-derived LOS was hydrolysed in 200 μL 1% acetic acid in milli-Q water (v/v) for 3 h at 100 °C with frequent vortexing. An equal volume of milli-Q water was added to the hydrolysate, which was then kept for 36 h at 4 °C and later centrifuged at 8,000 × g and 4 °C. The supernatant, containing the oligosaccharide (OS), was lyophilized. NMR samples were prepared in 99.9% D_2O buffer, containing 5 mM sodium phosphate, pD 7.2 (uncorrected value), and 200 mM NaCl.

All NMR spectra (Fig. S3-S11) were acquired at 310 K on a 600 MHz Bruker Avance spectrometer equipped with a cryoprobe, and processed with TopSpin 3.0 software (Bruker). The ^{1}H chemical shifts were referenced to the residual water signal using the equation δ (ppm) = 5.051 − 0.011 × T (°C)[18]. The ^{1}H and ^{13}C NMR OS spectra were assigned using a combination of TOCSY (*dipsi2phpr*), ^{1}H-^{13}C HSQC (*hsqcedetgp*) and ROESY (*roesyphpr*) of standard pulse sequences included in Bruker TOPSPIN software. TOCSY experiments were performed with 20 and 70 ms mixing times. ROESY experiments were performed in the phase sensitive mode with presaturation, and the spin-lock module was of 300 ms.

For STD experiments, the efficiency of on-resonance frequencies of δ = 7 ppm and -0.5 ppm was first compared, yielding identical STD patterns (Supplementary Fig. S9). As aromatic irradiation (7 ppm) resulted in higher protein saturation (82% vs 70% for aliphatic irradiation) this on-resonance frequency was used for the full set of STD NMR experiments.

Molecular dynamics simulations. The structures of Galα(1,4)Galβ (galabiose) and Galα(1,4)Galβ(1,4)Glcβ (globotriose) were built using the Carbohydrate Builder tool available at GLYCAM-Web[19], and minimized and parametrized for AMBER 12[20], MD simulations using GLYCAM 6 Force Field[21]. Protein–sugar complexes were built by docking the ligands into the Tyr-site of the crystal structure of the VAA–galactose complex (PDB code 1OQL). The structures of the two complexes were then processed with the XLEAP Amber module to get the input files for MD simulations. For the protein, the Force-Field 99 (ff99) was used[22]. A truncated octahedral box with dimensions of 10.0 Å for explicit TIP3P water molecules was defined[23]. MD simulations with no restraints in an explicit water solvent were carried out using the SANDER module in AMBER, with periodic boundary conditions and the particle-mesh Ewald approach[24] to account for electrostatic interactions. The protocol included four steps: 1) initial minimisation with protein and carbohydrate fixed, to allow water molecules to place properly, 2) minimisation of the whole system, 3) a 20-ps MD simulation with the position restrained for the complex, to relax the location of solvent molecules and to heat the system, and 4) a 10-ns unrestrained MD simulation of the complex at 300 K and 1 atm, with 50,600 structures saved for further analysis. The final frames were processed and analysed for robustness and equilibrium throughout the simulation, characterising its rmsd, potential, kinetic and total energies, temperature, pressure, volume, and density. Frames were clustered using the PTRAJ AMBER module to determine structure populations within the MD simulation. DBSCAN (density based) clustering algorithm was used, with a minimum of 30 points to make a cluster, 0.7 as the distance cut-off for forming clusters, and RMSD of atoms as distance metric. A total of 26 clusters for galabiose and 29 clusters for globotriose were obtained. The most representative structure for each cluster was selected for discussion.

REFERENCES

1 Stirpe, F. Ribosome-inactivating proteins. *Toxicon* **44**, 371-383, https://doi.org/10.1016/j.toxicon.2004.05.004 (2004).

2 Jiménez, M., André, S., Siebert, H.-C., Gabius, H.-J. & Solís, D. AB-type lectin (toxin/agglutinin) from mistletoe: differences in affinity of the two galactoside-binding Trp/Tyr-sites and regulation of their functionality by monomer/dimer equilibrium. *Glycobiology* **16**, 926-937, http://dx.doi.org/10.1093/glycob/cwl017 (2006).

3 Sweeney, E. C. *et al.* Preliminary crystallographic characterization of ricin agglutinin. *Proteins: Structure, Function, and Bioinformatics* **28**, 586-589, http://dx.doi.org/10.1002/(SICI)1097-0134(199708)28:4<586::AID-PROT12>3.0.CO;2-C (1997).

4 Lee, C.-H. & Tsai, C.-M. Quantification of bacterial lipopolysaccharides by the Purpald assay: measuring formaldehyde generated from 2-keto-3-deoxyoctonate and heptose at the inner core by periodate oxidation. *Analytical Biochemistry* **267**, 161-168, http://dx.doi.org/10.1006/abio.1998.2961 (1999).

5 Hood, D. W. *et al.* Sialic acid in the lipopolysaccharide of *Haemophilus influenzae:* strain distribution, influence on serum resistance and structural characterization. *Molecular Microbiology* **33**, 679-692, http://dx.doi.org/10.1046/j.1365-2958.1999.01509.x (1999).

6 Bouchet, V. *et al.* Host-derived sialic acid is incorporated into *Haemophilus influenzae* lipopolysaccharide and is a major virulence factor in experimental otitis media. *Proceedings of the National Academy of Sciences of the United States of America* **100**, 8898-8903, http://dx.doi.org/10.1073/pnas.1432026100 (2003).

7 Risberg, A. *et al.* Structural analysis of the lipopolysaccharide oligosaccharide epitopes expressed by a capsule-deficient strain of *Haemophilus influenzae* Rd. *European Journal of Biochemistry* **261**, 171-180, http://dx.doi.org/10.1046/j.1432-1327.1999.00248.x (1999).

8 Månsson, M., Hood, D. W., Moxon, E. R. & Schweda, E. K. H. Structural diversity in lipopolysaccharide expression in nontypeable *Haemophilus influenzae*. *European Journal of Biochemistry* **270**, 610-624, http://dx.doi.org/10.1046/j.1432-1033.2003.03399.x (2003).

9 Fox, K. L. *et al.* Identification of a bifunctional lipopolysaccharide sialyltransferase in *Haemophilus influenzae*: incorporation of disialic acid. *Journal of Biological Chemistry* **281**, 40024-40032, http://dx.doi.org/10.1074/jbc.M602314200 (2006).

10 Sonnenburg, J. L., van Halbeek, H. & Varki, A. Characterization of the acid stability of glycosidically linked neuraminic acid: Use in detecting de-*N*-acetyl-gangliosides in human melanoma. *Journal of Biological Chemistry* **277**, 17502-17510, http://dx.doi.org/10.1074/jbc.M110867200 (2002).

11 Spichtig, V., Michaud, J. & Austin, S. Determination of sialic acids in milks and milk-based products. *Analytical Biochemistry* **405**, 28-40, http://dx.doi.org/10.1016/j.ab.2010.06.010 (2010).

12 Barrientos, A. G. *et al.* Modulating glycosidase degradation and lectin recognition of gold glyconanoparticles. *Carbohydrate Research* **344**, 1474-1478, http://dx.doi.org/10.1016/j.carres.2009.04.029 (2009).

13 Jiménez, M., André, S., Gabius, H.-J. & Solís, D. Calibration of colorimetric protein assays for quantitation of plant AB toxins. *Analytical Biochemistry* **284**, 418-420, http://dx.doi.org/10.1006/abio.2000.4721 (2000).

14 Kalograiaki, I. *et al.* Combined Bacteria Microarray and Quartz Crystal Microbalance approach for exploring glycosignatures of nontypeable *Haemophilus influenzae* and recognition by host lectins. *Analytical Chemistry* **88**, 5950-5957, http://dx.doi.org/10.1021/acs.analchem.6b00905 (2016).

15 Lam, J. S., Anderson, E. M. & Hao, Y. in *Pseudomonas Methods and Protocols* (eds Alain Filloux & Juan-Luis Ramos) 375-402 (Springer New York, 2014).

16 Schägger, H. & Von Jagow, G. Tricine-sodium dodecyl sulfate-polyacrylamide gel electrophoresis for the separation of proteins in the range from 1 to 100 kDa. *Analytical biochemistry* **166**, 368-379 (1987).

17 Schägger, H. Tricine–SDS-PAGE. *Nature Protocols* **1**, 16, http://dx.doi.org/10.1038/nprot.2006.4 (2006).

18 Gottlieb, H. E., Kotlyar, V. & Nudelman, A. NMR chemical shifts of common laboratory solvents as trace impurities. *Journal of Organic Chemistry* **62**, 7512-7515, http://dx.doi.org/10.1021/jo971176v (1997).

19 Woods Group. *GLYCAM Web*, (2005-2017).

20 Case, D. A. *et al. AMBER 12*, University of California, San Francisco, (2012).

21 Kirschner, K. N. *et al.* GLYCAM06: A generalizable biomolecular force field. Carbohydrates. *Journal of Computational Chemistry* **29**, 622-655, http://dx.doi.org/10.1002/jcc.20820 (2008).

22 Wang, J., Cieplak, P. & Kollman, P. A. How well does a restrained electrostatic potential (RESP) model perform in calculating conformational energies of organic and biological molecules? *Journal of Computational Chemistry* **21**, 1049-1074, http://dx.doi.org/10.1002/1096-987X(200009)21:12<1049::AID-JCC3>3.0.CO;2-F (2000).

23 Jorgensen, W. L., Chandrasekhar, J., Madura, J. D., Impey, R. W. & Klein, M. L. Comparison of simple potential functions for simulating liquid water. *Journal of Chemical Physics* **79**, 926-935 (1983).

24 Mahoney, M. W. & Jorgensen, W. L. A five-site model for liquid water and the reproduction of the density anomaly by rigid, nonpolarizable potential functions. *Journal of Chemical Physics* **112**, 8910-8922, http://dx.doi.org/10.1063/1.481505 (2000).

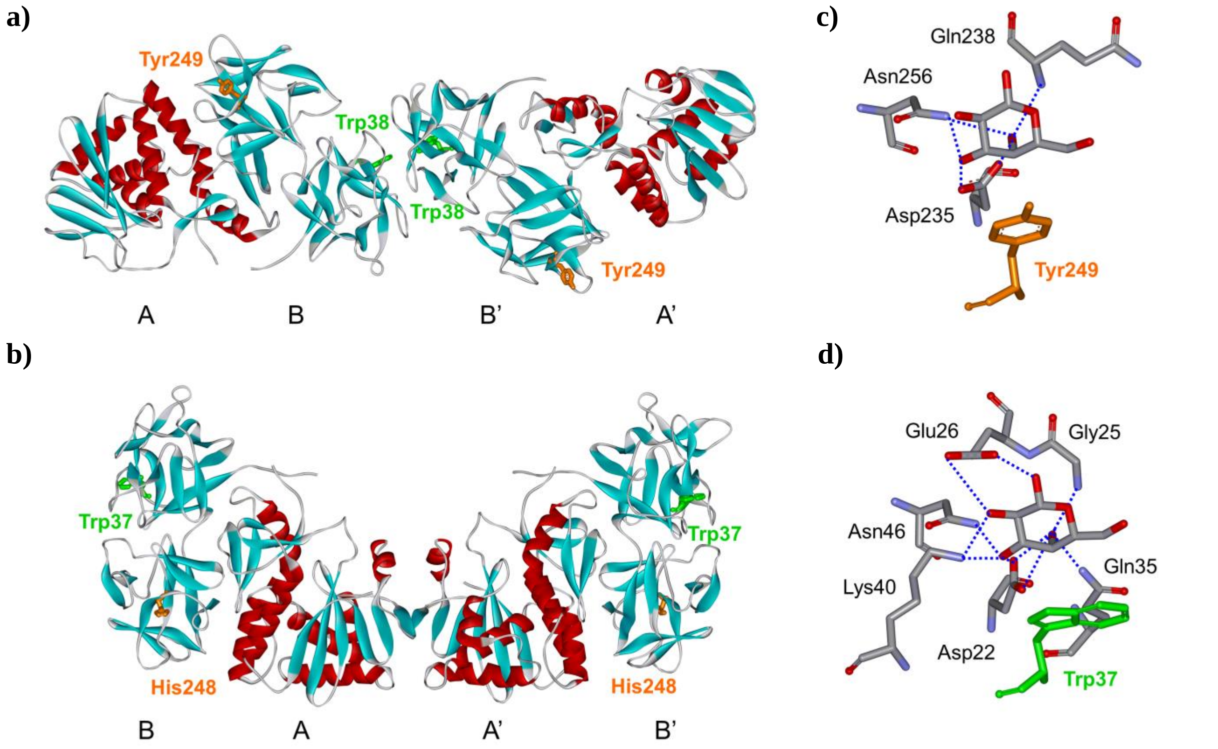

Figure S1. Overall topology of the VAA and RCA dimers and architecture of the operative binding sites. The side chains of Trp38/37 (coloured in green) and Tyr249 (coloured in orange) indicate the position of the carbohydrate-binding sites in the VAA (**a**) and RCA (**b**) dimers. In RCA, the Tyr site is not functional due to a mutation of the Tyr residue with His (coloured in orange). (**c**,**d**) Binding mode at the VAA Tyr site (**c**) and RCA Trp site (**d**) in the crystal structure of the agglutinin−galactose complexes. The main hydrogen bonds are shown as dotted blue lines. Structure files used were 1OQL and 1RZO.

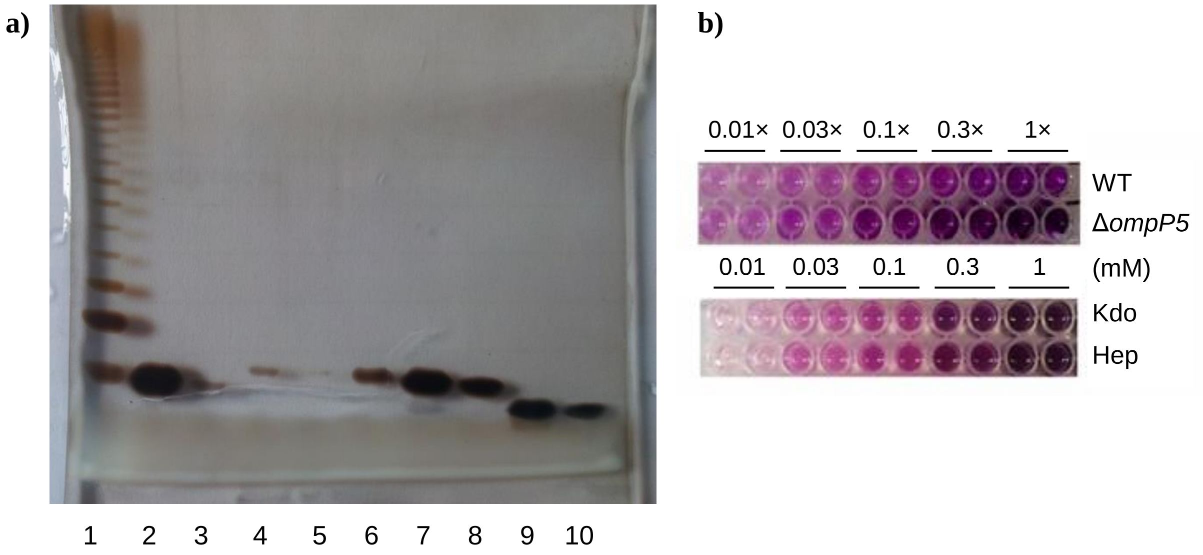

Figure S2. Electrophoretic analysis and colorimetric quantitation of purified LOSs. (**a**) DOC-PAGE gel illustrating the different electrophoretic mobility of the purified LOSs molecules. Samples of LOSs extracted and finally resuspended in 200 µL of distilled water were subjected to sodium deoxycholate-polyacrylamide gel electrophoresis, and LOS bands were visualized by silver staining (see Supplementary Methods). As reference of mobility and staining sensitivity, 1 µg of the Smooth form of *Salmonella minnesota* lipopolysaccharide (1), and 0.25 µg (2) and 50 ng (3) of the Rough b-form of the same lipopolysaccharide were also run. 4 and 5: 7.5 µL and 2 µL, respectively, of wild type NTHi375 LOS; 6: 1 µL of a 1:100 dilution of NTHi375Δ*ompP5* LOS; 7 and 8, 1 µL and 0.6 µL, respectively, of a 1:200 dilution of $LOS_{NTHi375\Delta \mathit{lgtF}}$; 9 and 10: 1 µL and 0.6 µL, respectively, of a 1:200 dilution of $LOS_{NTHi375\Delta \mathit{lpsA}}$. Please note that band shadows are visible. (**b**) Colorimetric quantitation by the Purpald assay. Differences in colour intensity for serial dilutions (1× to 0.01×) of a batch of NTHi375 WT and Δ*ompP5* LOS are illustrated. Kdo and L-glycero-D-manno-heptose at 0.01 to 1 mM were used as standards.

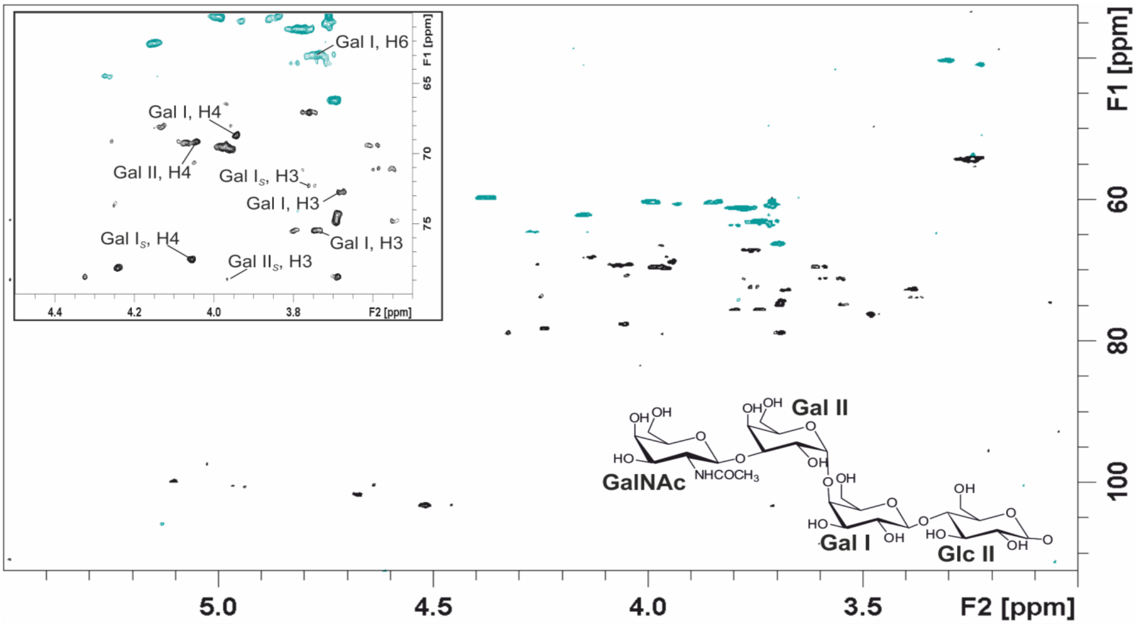

Figure S3. Section of the multiplicity-edited ^{1}H-^{13}C HSQC spectrum of LOS$_{RdKW20}$-derived oligosaccharides. The inset shows a spectral zoom at δ ~3.5-4.5 ppm (^{1}H, F2 axis) corresponding to a zone of special interest for galactose moieties (labelled for relevant protons). *S* stands for substituted residues. Chemical shifts and coupling constants of anomeric protons are listed in Table S1. CH_2 are shown in petrol; CH and CH_3 in black.

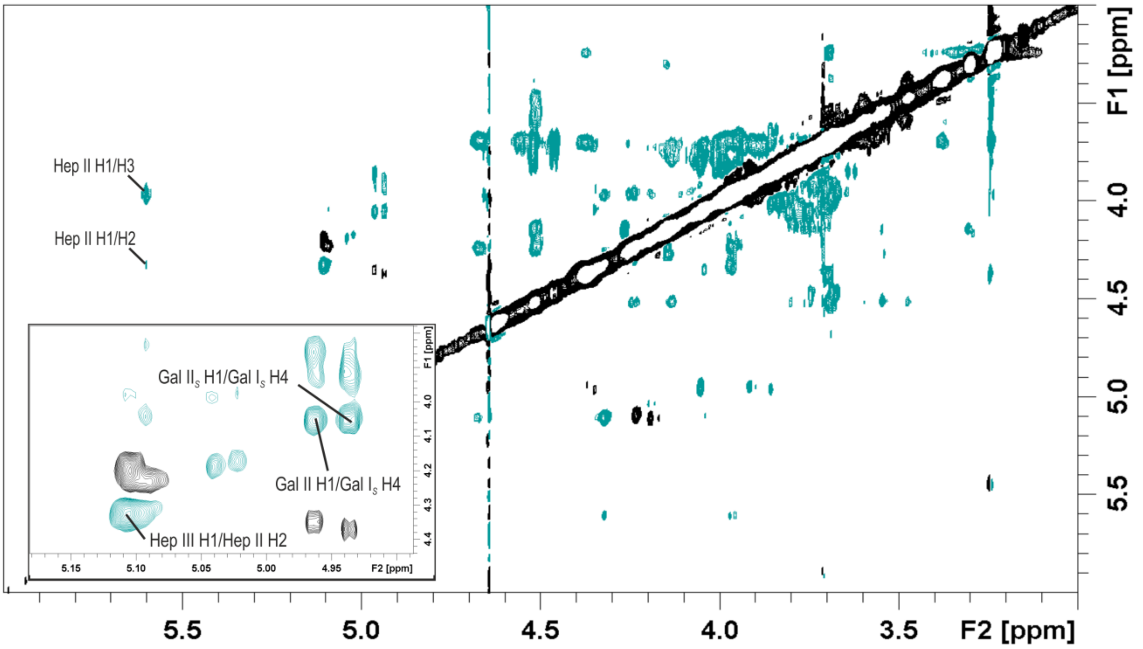

Figure S4. **Section of a ROESY spectrum of LOS$_{RdKW20}$-derived oligosaccharides.** The spectrum was acquired with a 300-ms spin-lock mixing time. Relevant intra- and inter-residue ROE contacts are labelled. In the zoomed section, *S* stands for substituted residues. Substitution of Gal II at position 3 results in a small but perceptible shift of the H3 resonance.

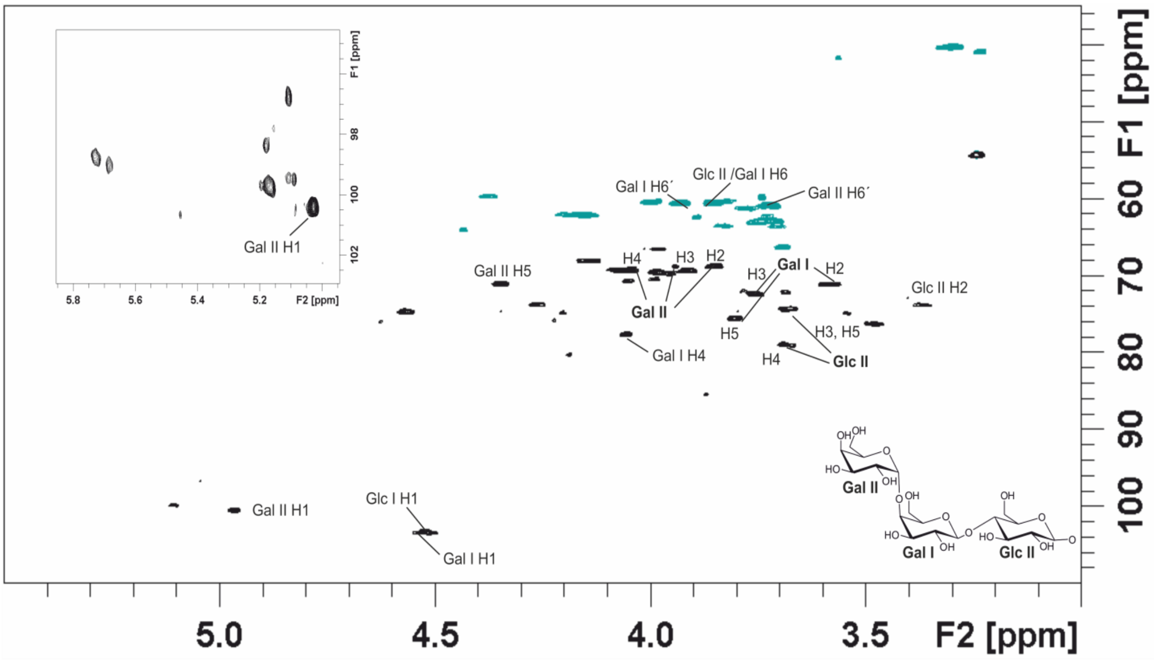

Figure S5. Section of the multiplicity-edited ^{1}H-^{13}C HSQC spectrum of $LOS_{NTHi375}$-derived oligosaccharides. Peak assignment is labelled for Hex4 glycoform protons and the anomeric proton of Glc I. Inset, α-Gal and Hep anomeric proton region (δ 5 to 6 ppm). Chemical shifts and coupling constants of anomeric protons are listed in Table S2. CH_2 are shown in petrol; CH and CH_3 in black.

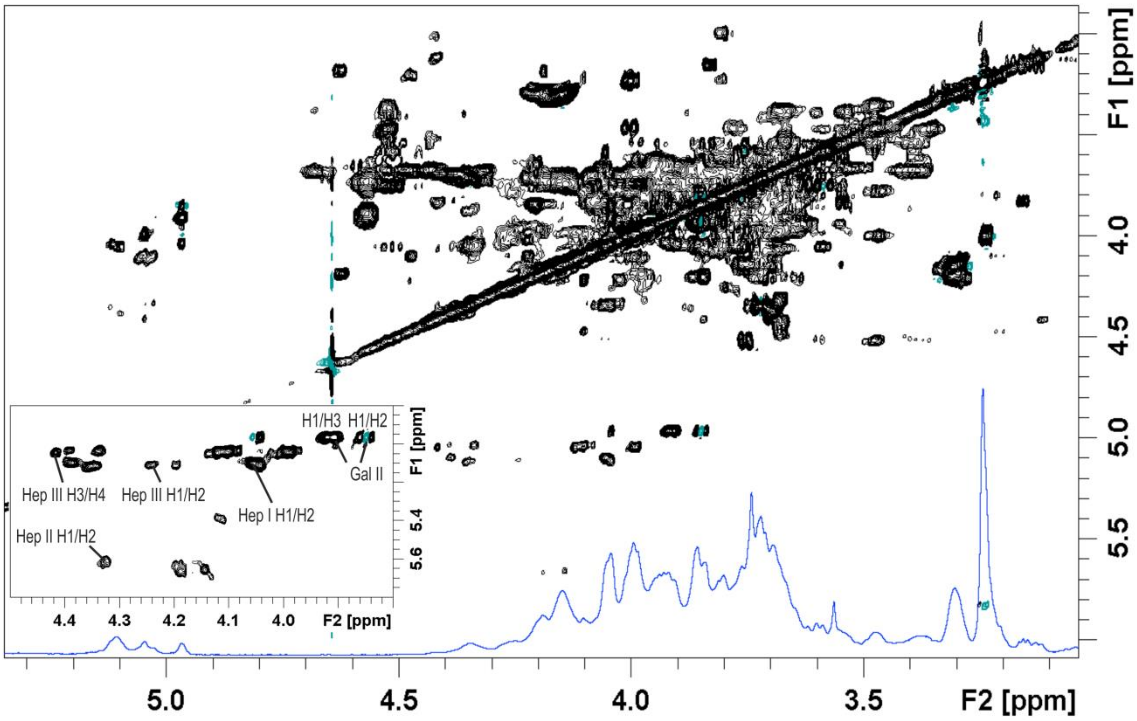

Figure S6. Section of the TOCSY spectrum of $LOS_{NTHi375}$-derived oligosaccharides. Mixing time was 70 ms. The corresponding 1D ^{1}H-spectrum was acquired applying a gradient-based water suppression pulse sequence (*zgesgp*). Relevant correlation peaks are labelled in the zoomed section.

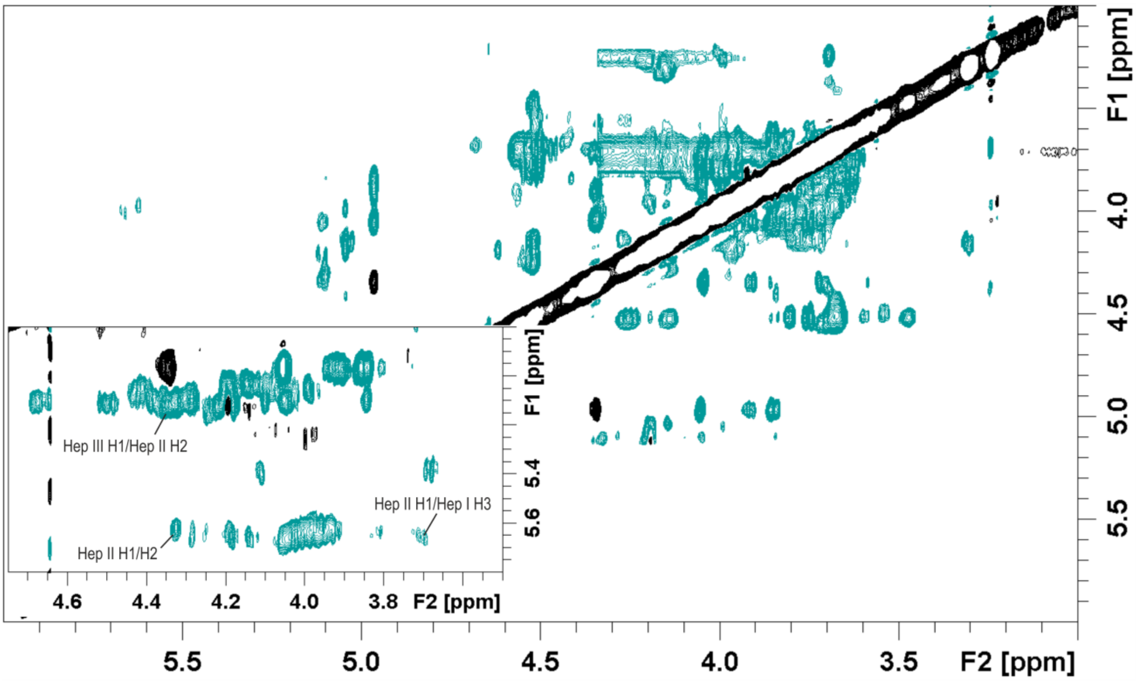

Figure S7. Section of a ROESY spectrum of $LOS_{NTHi375}$-derived oligosaccharides (I). The spectrum was acquired with a 300-ms spin-lock mixing time. In the zoomed section, relevant intra- and inter-residue ROE contacts are labelled, including transglycosidic ROE cross-peaks between proton pairs Hep III H1/Hep II H2 and Hep II H1/Hep I H3 (see also Table S3).

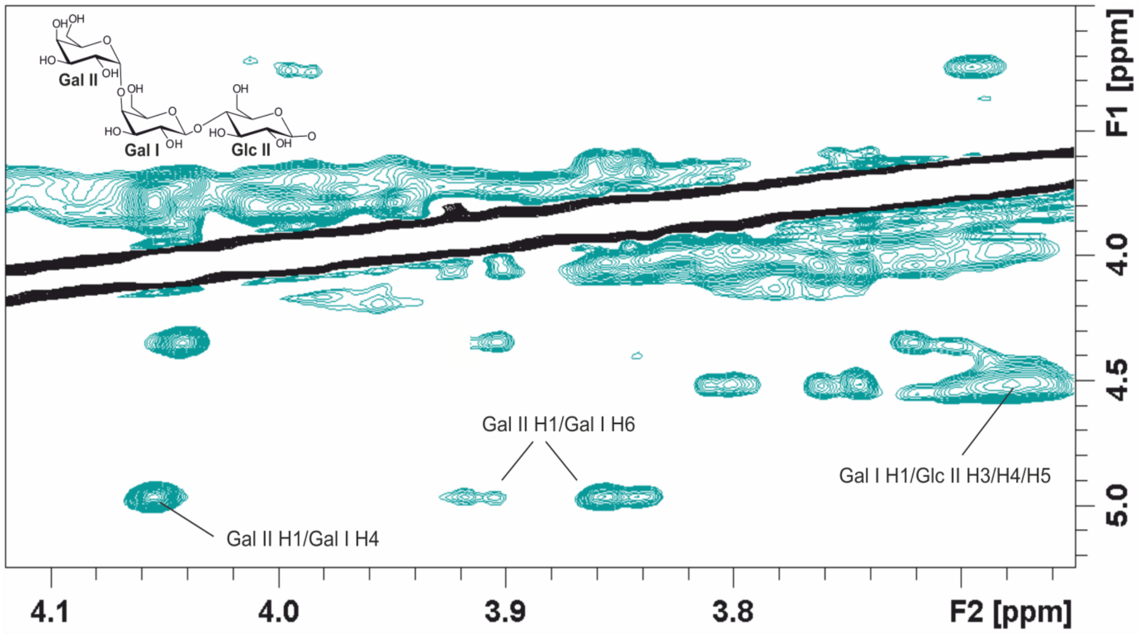

Figure S8. Section of a ROESY spectrum of $LOS_{NTHi375}$-derived oligosaccharides (II). The spectrum was acquired with a 300-ms spin-lock mixing time. Relevant inter-residue ROE contacts were observed between proton pairs Gal I H1/Glc II H3/H4/H5 and Gal II H1/Gal I H4/H6 (see also Table S3).

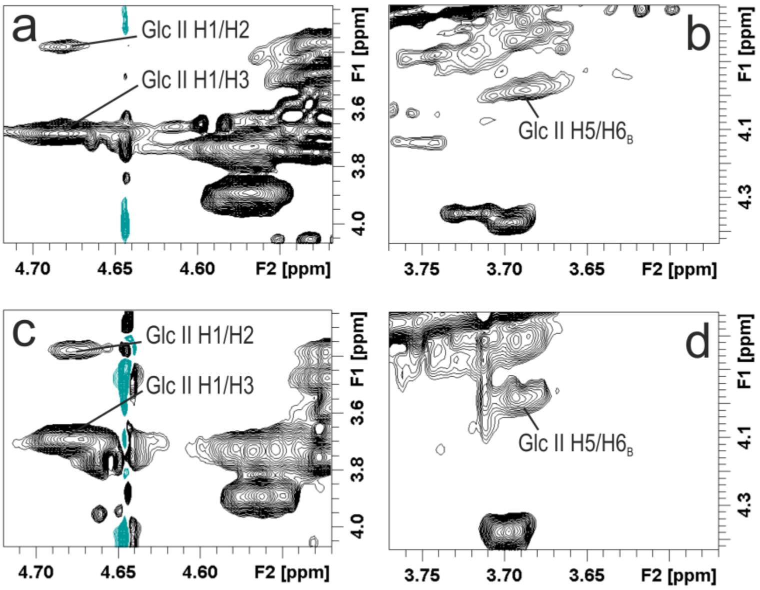

Figure S9. Comparison of sections of the TOCSY spectra of oligosaccharides derived from $LOS_{NTHi375}$ and LOS_{RdKW20}. The spectra of $LOS_{NTHi375}$- (**a**, **b**) and LOS_{RdKW20}-derived (**c**, **d**) oligosaccharides were acquired with a 70 ms mixing time. Relevant cross-peaks for Glc II are annotated.

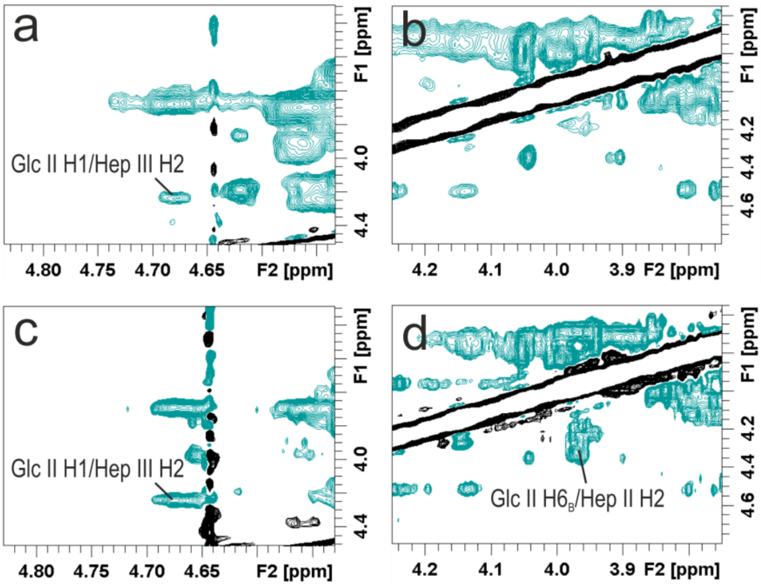

Figure S10. Comparison of sections of the ROESY spectra of oligosaccharides derived from $LOS_{NTHi375}$ and LOS_{RdKW20}. The spectra of $LOS_{NTHi375}$- (**a**, **b**) and LOS_{RdKW20}-derived (**c**, **d**) oligosaccharides were acquired with a 300 ms spin-lock mixing time. Strong inter-residual ROE contacts between the proton pairs Glc II H1/ Hep III H2 were common to oligosaccharide spectra from both strains (**a**, **c**). ROE contacts between Glc II H6 and Hep II H2 could be observed for LOS_{RdKW20}-derived OSs (**d**), while no crosspeak was spotted for NTHi375 (**b**).

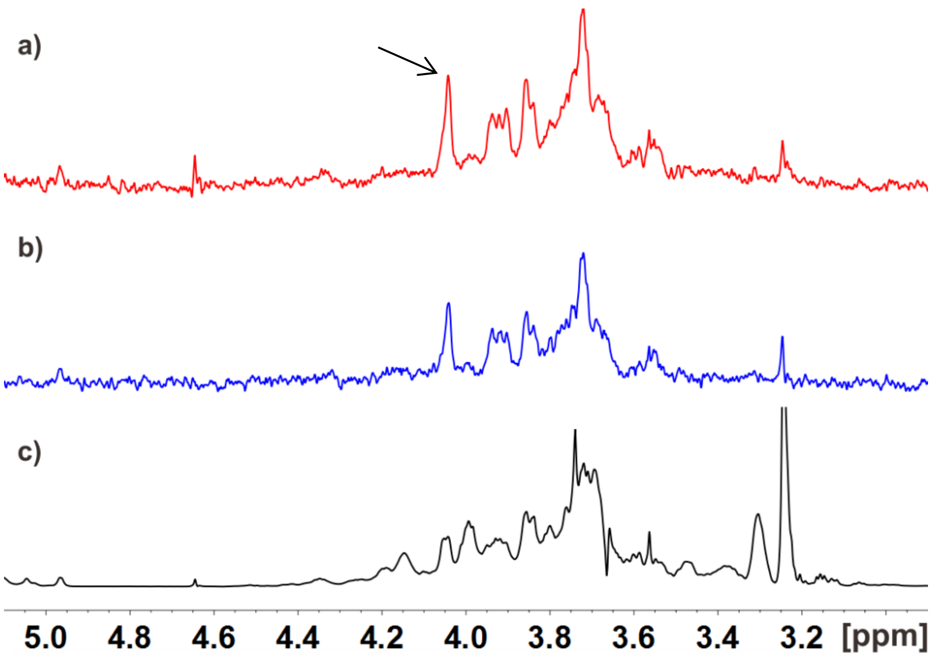

Figure S11. STD spectra obtained for the VAA–LOS$_{NTHi375}$-derived oligosaccharide complex. (**a**) Difference spectrum (STD) obtained upon irradiation at 7 ppm (scaled 128×). The most intense STD signal, corresponding to α-Gal H4, is indicated by an arrow. (**b**) Difference spectrum (STD) obtained upon irradiation at -0.5 ppm (scaled 128×). (**c**) *Off-resonance* spectrum obtained upon irradiation at 100 ppm (not scaled). All spectra were acquired at 310 K, referenced to the residual HDO signal, and illustrated upon subtraction of the protein signals.

Table S1. ^{1}H-^{13}C NMR chemical shifts for LOS$_{RdKW20}$-derived Hex3, Hex4, and Hex5 oligosaccharide glycoforms.

Isoform	Residue	Sugar unit	$^{3}J_{H,H}$†	H1<br>(C1)	H2<br>(C2)	H3<br>(C3)	H4<br>(C4)	H5<br>(C5)	H6A<br>(C6)	H6B	H7A<br>(C7)	H7B
all	Hep I	3,4)-l-α-D-Hep*p*-(1→	N/R	5.09-5.14<br>(99.7)	4.04<br>(69.1)	N/D	4.24*<br>(78.1)	N/D	4.14*<br>(64.5)	N/D	N/D	N/D
all	Hep II	2)-l-α-D-Hep*p*-(1→	N/R	5.60-5.68<br>(99.2)	4.32 [4.19]<br>(78.8)	3.97<br>(78.9)	3.68<br>(72.8)	N/D	N/D	N/D	3.75<br>(65.0)	3.92<br>(61.2)
all	Hep III	2)-l-α-D-Hep*p*-(1→	N/R	5.10-5.13<br>(99.7)	4.24 [4.19]<br>(78.1)	3.98<br>N/D	3.80<br>(75.4)	N/D	N/D	N/D	N/D	N/D
all	Glc I	β-D-Glc*p*-(1→	8.2	4.52<br>(103.3)	3.38<br>(73.8)	3.48<br>(76.2)	3.55<br>(74.8)	3.61*<br>(74.8)	4.14<br>(64.5)	4.27<br>(64.5)		
all	Glc II	4)-β-D-Glc*p*-(1→	7.5	4.67<br>(101.6)	3.38<br>(72.6)	3.69<br>(74.3)	3.69<br>(78.8)	3.69<br>(74.6)	3.85<br>(60.2)	3.98<br>(60.2)		
Hex3	Gal I	β-D-Gal*p*-(1→	7.7	4.47<br>(103.1)	3.55<br>(73.7)	3.68<br>(72.7)	3.94<br>(68.7)	3.74<br>(75.5)	3.78<br>(61.2)	N/D		
Hex4	Gal I	4)-β-D-Gal*p*-(1→	7.5	4.52<br>(103.3)	3.60<br>(71.1)	3.76<br>(72.3)	4.05<br>(77.6)	3.79*<br>(75.5)	3.86<br>(60.5)	3.92<br>(60.5)		
Hex4	Gal II	α-D-Gal*p*-(1→	3.5	4.96<br>(100.4)	3.86<br>(68.7)	3.90<br>N/D	4.04<br>(69.1)	4.35*<br>(71.0)	3.71-2<br>(60.7)			
Hex5	Gal II	3)-l-α-D-Gal*p*-(1→	3.7	4.94<br>(100.6)	3.90<br>N/D	3.96<br>(78.9)	4.26<br>(69.0)	N/D	N/D			
Hex5	GalNAc	β-D-GalNAc*p*-(1→	N/R	4.64<br>(100.2)	3.95<br>(68.7)	3.90<br>(69.3)	4.04<br>(69.2)	3.69*<br>(71.0)				
all	PEtn			4.16<br>(62.1)	3.22<br>(40.8)							
all	PCho			4.38<br>(59.6)	3.69<br>(66.2)							

$^{3}J_{H,H}$ values for anomeric ^{1}H resonances (H1) were calculated from TOCSY spectra. Unless otherwise stated, chemical shifts are the same (± 0.02 ppm) for the conserved inner-core residues, shared by all glycoforms. N/R: not resolved, N/D: not defined. *Tentatively assigned from ROESY spectra.

Table S2. ^{1}H-^{13}C NMR chemical shifts for LOS$_{NTHi375}$-derived Hex3 and Hex4 oligosaccharide glycoforms.

Isoform	Residue	Sugar unit	$^{3}J_{H,H}$	H1 (C1)	H2 (C2)	H3 (C3)	H4 (C4)	H5 (C5)	$H6_A$ (C6)	$H6_B$	$H7_A$ (C7)	$H7_B$
all	Hep I	3,4)-l-α-D-Hep*p*-(1	N/R	5.04-5.11 (96.7-100.5)	4.03-4.05 (70.6)	3.99 (N/D)	4.25* (78.1)	N/D	4.14 (64.5)	4.24-4.26 (64.5)	N/D	N/D
all	Hep II	2)-l-α-D-Hep*p*-(1	3.8	5.60-5.68 (98.8-99.2)	4.32 [4.14] (78.8)	3.98 (N/D)	N/D	N/D	4.56 (74.6)		3.73 (62.3)	3.89 (62.3)
all	Hep III	2)-l-α-D-Hep*p*-(1	N/R	5.10-5.13 (99.7)	4.25 (78.2)	5.04 (73.3)	4.41 (70.2)	N/D	N/D	N/D	N/D	N/D
all	Glc I	β-D-Glc*p*-(1	8.2	4.52 (103.3)	3.38 (73.8)	3.48 (76.2)	3.55 (74.8)	3.60* (74.9)	4.14 (64.5)	4.26 (64.5)		
all	Glc II	4)-β-D-Glc*p*-(1	7.5	4.68 (101.8)	3.38 (72.7)	3.69 (74.3)	3.69 (78.8)	3.69 (74.6)	3.84 (60.5)	3.97 (60.5)		
Hex3	Gal I	β-D-Gal*p*-(1	N/R	4.47 (103.1)	3.55 (74.8)	3.68 (73.3)	3.94 (68.7)	3.75 (75.5)	3.74 (62.8)	N/D		
Hex4	Gal I	4)-β-D-Gal*p*-(1	7.5	4.53 (103.3)	3.59 (70.7)	3.76 (72.3)	4.06 (77.6)	3.80* (75.5)	3.86 (60.5)	3.92 (60.5)		
Hex4	Gal II	α-D-Gal*p*-(1	3.7	4.97 (102.1)	3.84 (68.7)	3.90 (69.3)	4.04 (70.6)	4.35* (71.0)	3.72 (60.8)			
all	PEtn			4.15 (62.1)	3.25 (40.8)							
all	PCho			4.38 (59.6)	3.69 (66.2)							

Hex3 bears Galβ(1,4)Glcβ(1,2) at Hep III, while Hex4 bears Galα(1,4)Galβ(1,4)Glc (1,2) at this position. $^{3}J_{H,H}$ values for anomeric ^{1}H resonances (H1) were calculated from TOCSY spectra. Unless otherwise stated, chemical shifts are the same (± 0.02 ppm) for the conserved inner-core residues, shared by both glycoforms. N/R: not resolved, N/D: not defined. *Tentatively assigned from ROESY spectra.

Table S3. Proton ROE data for $LOS_{NTHi375}$-derived oligosaccharides

Anomeric proton	Observed proton ROE	
	Intra-residue	Inter-residue
5.04-5.11 (Hep I)	4.03-4.05 (H2)	–
5.60-5.68 (Hep II)	4.32; 4.14 (H2)	3.99 (Hep I H3); 4.25 (Hep III H2)
5.10-5.13 (Hep III)	4.25 (H2)	5.60-5.68 (Hep II H1); 4.32 (Hep II H2); 4.68 (Glc II H1)
4.52 (Glc I)	3.48 (H3)[a] and 3.60 (H5)[a]	4.25 (Hep I H4); 4.14 ($H6_A$)
4.68 (Glc II)	3.69 (H3/H5)[b]	5.10-5.13 (Hep III H1); 4.25 (H2)
4.53 (Gal I)	3.76 (H3) and 3.80 (H5)	3.69 (Glc II H4)[b]
4.97 (Gal II)	3.84 (H2) and 3.90 (H3)	4.06 (Gal I H4); 3.86 ($H6_A$); 3.92 ($H6_B$)

[a] Similar intra-residue distance of ~2.5 Å from the anomeric proton; [b] ^{1}H-signals corresponding to H3, H4 and H5 of Glc II overlapped (~3.69 ppm), resulting in broadened ROE cross-peaks.

Table S4. Water-mediated contacts established by VAA with Galα(1,4)Galβ and Galα(1,4)Galβ(1,4)Glcβ observed through the MD simulations

Sugar unit	Sugar atom	Protein residue:atom	Number of clusters[a]	Sugar atom	Protein residue:atom	Number of clusters[a]
**Galα(1,4)Galβ**						
Gal II	O2	TYR249:OH	1/26	H2O	TYR249:OH	2/26
		THR252:OG1	1/26		THR252:HG1	1/26
		LYS254:HZ1	1/26		LYS254:HZ3	2/26
		LYS254:HZ2	1/26		ASN256:OD1	7/26
		ASN256:OD1	1/26			
	O3	ASP235:OD1	9/26			
		THR252:HG1	1/26			
		LYS254:HZ1	1/26			
		LYS254:HZ2	1/26			
		LYS254:HZ3	1/26			
	O5	GLN238:HE22	1/26			
	O6	SER200:HG	4/26	H6O	SER200:HG	3/26
		GLN238:H	1/26		GLN238:H	2/26
		GLN238:HE22	2/26		GLN238:HE22	2/26
		TYR249:HH	2/26		TYR249:HH	1/26
Gal I	O1	SER200:HG	1/26			
		TYR249:OH	3/26			
	O2	SER200:HG	1/26	H2O	TYR249:HH	4/26
		TYR249:OH	1/26			
	O3	GLN238:HE22	2/26	H3O	GLN238:HE22	1/26
					TYR249:HH	1/26
	O5	GLN238:HE22	1/26			
		TYR249:OH	2/26			
		LYS254:HZ1	1/26			
	O6	GLN238:HE22	1/26	H6O	GLN238:O	1/26
					TYR249:HH	1/26
					LYS254:HZ3	1/26

Table S4 (continued)

Sugar unit	Sugar atom	Protein residue:atom	Number of clusters[a]	Sugar atom	Protein residue:atom	Number of clusters[a]
**Galα(1,4)Galβ(1,4)Glcβ**						
Gal II	O2	ASP235:OD1	1/29	H2O	GLN238:O	1/29
		LYS254:HZ1	2/29		THR252:HG1	1/29
		LYS254:HZ2	3/29		LYS254:HZ1	3/29
		LYS254:HZ3	4/29		LYS254:HZ2	1/29
		ASN256:OD1	3/29		ASN256:OD1	14/29
	O3	ASP235:OD1	6/29			
		THR252:HG1	4/29			
		LYS254:HZ2	1/29			
		LYS254:HZ3	2/29			
		ASN256:OD1	13/29			
	O6	SER200:HG	9/29	H6O	SER200:HG	1/29
		GLN238:HE22	2/29		GLN238:HE22	1/29
		TYR249:HH	1/29		GLN238:OE1	1/29
					TYR249:HH	1/29
Gal I	O2	TYR249:HH	3/29	H2O	GLN238:HE21	1/29
					TYR249:HH	1/29
	O3	TYR249:HH	3/29	H3O	TYR249:HH	2/29
	O5	TYR249:OH	1/29			
	O6	LYS254:HZ2	1/29			
Glc	O2	LYS254:HZ3	1/29	H2O	TYR249:OH	1/29
	O3	TYR249:HH	1/29	H3O	TYR249:OH	2/29
		TYR249:OH	2/29		LYS254:HZ3	1/29
		LYS254:HZ1	1/29			
		LYS254:HZ2	2/29			
	O4	TYR249:HH	2/29			

[a] Clusters out of 26 for Galα(1,4)Galβ and of 29 for Galα(1,4)Galβ(1,4)Glcβ in which the contact was detected.
